# The effects of the ethanol extract of *Cordia myxa* leaves on the cognitive function in mice

**DOI:** 10.1186/s12906-022-03693-z

**Published:** 2022-08-10

**Authors:** Gülsen Kendir, Ho Jung Bae, Jihyun Kim, Yongwoo Jeong, Hyo Jeoung Bae, Keontae Park, Xingquan Yang, Young-jin Cho, Ji-Young Kim, Seo Yun Jung, Ayşegül Köroğlu, Dae Sik Jang, Jong Hoon Ryu

**Affiliations:** 1 Department of Pharmaceutical Botany, Faculty of Pharmacy, Süleyman Demirel University, Isparta, Turkey; 2 Department Pharmaceutical Botany, Faculty of Pharmacy, İstinye University, İstanbul, Turkey; 3 Agriculture and Life Science Research Institute, Kangwon National University, Gangwon-do, Chuncheon, 24341 Republic of Korea; 4 Department of Life and Nanopharmaceutical Science, Kyung Hee University, Seoul, 02447 Republic of Korea; 5 Department of Biomedical and Pharmaceutical Sciences, Kyung Hee University, Seoul, 02447 Republic of Korea; 6 Department of Pharmaceutical Botany, Faculty of Pharmacy, Ankara University, Tandogan, Ankara, Turkey; 7 Department of Pharmaceutical Botany, Faculty of Pharmacy, Afyonkarahisar Health Sciences University, Afyon, Turkey; 8 Department of Oriental Pharmaceutical Science, College of Pharmacy, Kyung Hee University, Kyunghee-daero 26, Dongdeamun-gu, Seoul, 02447 Republic of Korea

**Keywords:** *Cordia myxa*, Alzheimer’s disease, Recognition memory, Sensorimotor gating, PI3K-Akt-GSK3β signaling, ERK-CREB signaling

## Abstract

**Background:**

*Cordia myxa* L. (Boraginaceae) is widely distributed in tropical regions and it’s fruits, leaves and stem bark have been utilized in folk medicine for treating trypanosomiasis caused by *Trypanosoma cruzi*. A population-based study showed that *T. cruzi* infection is associated with cognitive impairments. Therefore, if *C. myxa* has ameliorating activities on cognitive function, it would be useful for both *T. cruzi* infection and cognitive impairments.

**Methods:**

In this study, we evaluated the effects of an ethanol extract of leaves of *C. myxa* (ELCM) on memory impairments and sensorimotor gating deficits in mice. The phosphorylation level of protein was observed by the Western blot analysis.

**Results:**

The administration of ELCM significantly attenuated scopolamine-induced cognitive dysfunction in mice, as measured by passive avoidance test and novel object recognition test. Additionally, in the acoustic startle response test, we observed that the administration of ELCM ameliorated MK-801-induced prepulse inhibition deficits. We found that these behavioral outcomes were related with increased levels of phosphorylation phosphatidylinositol 3-kinase (PI3K), protein kinase B (Akt) and glycogen synthase kinase 3 beta (GSK-3β) in the cortex and extracellular signal-regulated kinase (ERK) and cAMP response element-binding protein (CREB) in the hippocampus by western blot analysis.

**Conclusions:**

These results suggest that ELCM would be a potential candidate for treating cognitive dysfunction and sensorimotor gating deficits observed in individuals with neurodegenerative diseases.

**Supplementary Information:**

The online version contains supplementary material available at 10.1186/s12906-022-03693-z.

## Introduction

Alzheimer’s disease (AD) is one of the most prevalent neurodegenerative diseases in developed countries. AD entered our daily life as an irreversible and fatal disease that gradually impairs memory, learning, thinking, judgment and communication skills [1]. The accumulation of amyloid β (Aβ) plaques and neurofibrillary tangles, phosphorylated tau proteins, and progressive brain atrophy are the most important pathological findings associated with the disease [2]. In addition, inflammation, oxidative damage, glutamate excitotoxicity and cholinergic loss are presumed to contribute to neurodegeneration in AD [3–5]. The etiology of AD remains unclear, and a proper cure is not available for this disease, although acetylcholinesterase (AChE) inhibitors or NMDA receptor antagonists are prescribed in clinic [6, 7]. These allopathic medicines act through one mechanism and have several adverse effects that must be overcome. Herbal extracts tend to provide some benefits due to their versatile mechanisms of action and low adverse effects [8]. Due to the potential of herbs, the number of studies in this area has been increasing over time.

*Cordia myxa* L. belongs to the Boraginaceae family, which is well known as the “Indian cherry and Assyrian plum” due to its edible fruits [9]. It is spread from the eastern Mediterranean region to eastern India, tropical Africa, tropical Asia and Australia [10]. The decoction form of the leaves is used to treat stomach, brain and breast cancer in Ghana and against cough and cold in India [11, 12]. It is known as “Yellim ağacı” or “Dıbık” in Turkey and grows only in Hatay Province of Turkey [13]. The antioxidant, antimicrobial, antidiabetic, cytotoxic, anti-inflammatory, analgesic, antipyretic and trypanocidal activities of *C. myxa* leaves were documented in *in vitro* studies [14–19]. Traditionally, *C. myxa* leaves have been used to treat trypanosomiasis, known as sleeping sickness [20]. *Trypanosoma cruzi-*induced trypanosomiasis in rats exhibited significant sleep impairments and spontaneous alternation behavior [21]. In addition, a population-based study showed that *T. cruzi* infection is associated with cognitive impairments, although the exact mechanism is unclear [22]. Therefore, we hypothesized that the active constituents of *C. myxa* leaves would penetrate into the brain and would be potential therapeutic agent if it ameliorates cognitive dysfunction. However, its function in cognition has not been investigated.

In this study, we aimed to evaluate the effects of an ethanol extract of leaves of *C. myxa* (ELCM) grown in Turkey on scopolamine-induced memory impairments in mice using several behavioral tasks. We also studied the effect of ELCM on MK-801-induced abnormalities in sensorimotor gating dysfunction. Scopolamine and MK-801 are nonselective muscarinic receptor antagonist and uncompetitive NMDA receptor antagonist, respectively. In addition, we tried to explore the mechanism of action of ELCM using Western blotting.

## Materials and methods

The protocol used in this study complied with relevant institutional, national and international guidelines and legislation required for animal and plant studies.

### Animals

ICR mice (male, 25–30 g) from Orient Bio Co., Ltd., a branch of Charles River Laboratories (Seongnam, Gyeonggi-do, Korea) were used for present study. The experimental animals were randomly divided and cured in groups of 5 per cage and provided food and water ad libitum. They were domesticated under a constant temperature (23 ± 1 °C) and humidity (60 ± 10%) on a 12 h light/dark cycle at university animal facility. All protocols in this study were approved by the Committee on the Ethics of Animal Experiments of Kyung Hee University (IACUC permit number: KHUASP(SE)-19–024), in compliance with the Animal Care Handbook published by the Kyung Hee University (NIH publication no.85–23, revised 1996) and ARRIVE guidelines. We used a total of 216 mice in this study as follows: passive avoidance test, *n* = 60; novel object recognition test, *n* = 60; acoustic startle response test, *n* = 60; western blot analysis, *n* = 36. Mice were randomly allocated for each experiment.

### Materials

All the materials used in the present study were purchased from common commercial sources and the highest grade available. Scopolamine, donepezil, rosmarinic acid, ascorbic acid, gallic acid, Folin-Ciocalteu reagent, MK-801 and aripiprazole were obtained from Sigma Aldrich (St. Louis, MO). All drugs and solutions were prepared freshly before the test. In 0.9% saline solution, scopolamine, donepezil and MK-801 were dissolved. ELCM and aripiprazole were dissolved in a 10% Tween80 solution. The sources of primary and secondary antibodies for Western blotting were described in the Supplementary materials (Supplementary Materials and Methods).

### Plant material and extraction

*C. myxa* leaves were picked in Hatay Province (Samandağ) in September 2018 and authenticated by M. Vural & S.T. Körüklü. The voucher specimens were deposited in the Ankara University Faculty of Pharmacy Herbarium (AEF 28,679). The leaves of *C. myxa* (50 g) were extracted using hot maceration with 2.5 L of ethanol (70% v/v) at 60 ℃ for 2 h. After the first extracts were obtained, the second extraction was conducted with 2 L of ethanol (70% v/v) using the same procedure. The obtained extracts (ELCM) were filtered, evaporated using a rotary evaporator and lyophilized. (yield: 11.06 ± 0.05%).

#### Determination of the total phenolic content

Gallic acid was used as a standard. Serial dilutions of gallic acid were prepared in the range of 50–800 μg/mL. The following processes were performed in 96-well plates, and the calibration plot were generated. The total phenolic content was measured as gallic acid equivalents and expressed in mg GAE/g dw extract (dw: dry weight) ± standard deviation mean (SD). The 20 μL of each extract (0.4 mg/mL) and 100 μL of Folin-Ciocalteu reagent were added to the wells, mixed well and incubated for 5 min. Then, 80 μL of a 7.5% sodium carbonate solution were added and mixed well. The plate was covered and incubated at room temperature for 2 h. The absorbances of the samples were measured at 750 nm using a spectrophotometric microplate reader (FLUOstar Omega (BMG Labtech)) [23].

#### DPPH radical scavenging assay

The DPPH (2,2-diphenyl-1-picrylhydrazyl radical) scavenging assay was performed in a 96-well microplate using a FLUOstar Omega microplate reader (BMG Labtech). Different concentrations (31.25–1000 μg/mL) of each extract were pipetted into a 96-well plate (2 μL). The DPPH solution (in ethanol) was added to each well (198 μL of a 200 μM solution). The mixture was incubated at room temperature in the dark for 30 min. The absorbance was read at 515 nm. Ascorbic acid was used as standard. DPPH radical scavenging capacities of samples were calculated using the following equation: % scavenging capacity = [(Ao − As)/Ao] * 100; Ao = the absorbance of the control (DPPH solution without sample) at 515 nm; As = the absorbance of different concentrations of the sample or reference incubated with DPPH at 515 nm. The antioxidant activity was reported as IC_50_ values. The results represent the means ± SD of at least 2 independent assays [24].

#### HPLC analysis

To ensure the consistency of herbal preparation, the quantitative analysis of the rosmarinic acid content in the 70% EtOH extract of *C. myxa* leaves was performed using Waters Alliance 2795 and Waters 996 PDA. MassLynx version 4.1 software operated the system control and was used for data analysis. The analytical chromatogram was developed using an Eclipse XD8-C18 column (Agilent Technologies, 150 × 4.6 mm I.D., 5 μm, Santa Clara, CA, USA). The mobile phase consisted of water containing 0.1% formic acid (solvent A) and acetonitrile containing 0.1% formic acid (solvent B) and isocratic elution was performed at a flow rate of 0.5 ml/min using the following conditions: 21% B (0–25 min) and 100% B (25–30 min). The column was equilibrated at the initial conditions for 15 min before the next injections. The injection volume was 10 μL, the detection wavelength was set to 330 nm, and the column temperature was 35 °C. Rosmarinic acid and the extract were diluted with methanol and filtered with a 0.20 μm syringe filter (Whatman Inc., Maidstone, UK) before the HPLC analysis. The analysis was repeated three times to check its reproducibility.

### Passive avoidance task

This test was performed with an apparatus that consisted of equal sized (20 × 20x20 cm) light and dark compartments separated by a guillotine door (5 × 5 cm). Learning and memory impairments were induced by the administration of scopolamine (1 mg/kg, i.p.). Donepezil (5 mg/kg, p.o.) was used as a positive control. Animals were administered with scopolamine 30 min before the acquisition trial. One hour after ELCM (150, 300 or 600 mg/kg, p.o.) or donepezil administration, the mouse was placed in the light compartment, and the door between the two compartments was opened after 10 s. When the mouse entered the dark compartment, the door was closed and an electrical foot shock (0.5 mA) of 3 s in duration was applied through the stainless steel rods. If the mouse did not enter the dark compartment within 60 s, it was introduced into the dark compartment, and the latency time was recorded as 60 s. The interval between the acquisition trial and the retention trial was 24 h. The same test was repeated after 24 h, and we analyzed whether the mice passed to the dark compartment in 300 s. The latency time was recorded as 300 s for the mice that did not pass into the dark compartment. If they passed to the dark compartment before this time, the latency time was recorded for each mouse [25, 26].

### Novel object recognition task

The novel object recognition test was conducted using a square open area composed of black polyvinyl plastic (25 × 25x25 cm). This test was composed of habituation, training and test sessions conducted over 3 days. During the habituation session, the mice were maintained in the experimental apparatus for 10 min without any objects. During the training session, two small objects were placed in the apparatus after 2 min of habituation, and the mice were allowed to explore them for 5 min. The test session was performed 24 h after the training session. In this session, the objects were placed into the apparatus after 2 min of habituation. Thereafter, one of the objects was replaced with a novel object, and the mice were allowed to explore the objects for 5 min. After each session, the apparatus and objects were cleaned with a 70% ethanol spray. One hour before the training session, ELCM (150, 300 or 600 mg/kg, p.o.) or donepezil was administered to the mice. The mice were also treated with scopolamine (1 mg/kg, i.p.) 30 min before the training session. In the test session, exploratory behavior of the mice was recorded with a video camera-based on an EthoVision system (Noldus, Wageningen, The Netherlands) for 5 min, and the time spent exploring each object (familiar object, T_familiar_; novel object, T_novel_) was analyzed by a researcher who had not any information for treatment groups. The discrimination ratio was calculated with the following formula, as described elsewhere [27]: (T_novel_-T_familiar_)/(T_novel_ + T_familiar_) × 100.

### Acoustic startle response and prepulse inhibition test

The acoustic startle response (ASR) and prepulse inhibition (PPI) tests were performed in SR-LAB startle compartments containing a Plexi-glass cylinder attached to the platform with a piezoelectric unit and a high-frequency loudspeaker (San Diego Instruments, San Diego, CA), as described in a previous study [28], with slight modification [29]. The high-frequency loudspeaker produced continuous background noise at 70 dB and various acoustic stimuli. The Plexi-glass cylinder estimated the whole body startle response of animals and the piezoelectric unit converted these responses to analog signals. And then, a computer digitized these signals and stored them. The acoustic startle response was calculated as the average response of the trials that were performed at 1 ms intervals from stimulus onset. The PPI test sessions consisted of non-stimulus (n.s.) trials, startle trials (pulse alone) and prepulse trials (prepulse + pulse) were started after the ASR test sessions. The n.s. trial included background noise only. The pulse alone trial was composed of constant noise (80, 90, 100, 110 or 120 dB) for 40 ms. Prepulse + pulse trials were composed of a 20 ms noise prepulse, 80 ms delay, and then a 120 dB startle pulse for 40 ms (100 ms onset to onset). The acoustic prepulse intensities were 73, 76, 82, and 86 dB, which were 3, 6, 12 and 16 dB above the background noise (70 dB). The mice were maintained at a background noise level of 70 dB for a 5 min acclimation period, and the noise persisted throughout the session. The both ends of test session had five repetitions of the pulse alone (120 dB) trial; in between, each acoustic or n.s. trial type was conducted 10 times in a pseudorandom order. The intertrial interval was an average of 21 s (range: 12–30 s) between trials. The MK-801 (0.2 mg/kg, i.p.) or aripiprazole (1 mg/kg, i.p.) were administered 30 min before the behavioral tasks and ELCM (150, 300 or 600 mg/kg, p.o.) or the vehicle was administered to the mice 1 h before each task. The amount of PPI was calculated as a percentage score for each acoustic prepulse trial type, as described previously [29].

### Western blot analysis

The mice were treated with ELCM (150, 300 or 600 mg/kg, p.o.) or donepezil (5 mg/kg, p.o.), and 30 min later, scopolamine (1 mg/kg, i.p.) was also treated. After 30 min from scopolamine-treated mice, the cortical and hippocampal tissues were isolated from both hemispheres for Western blot analysis. The Tris–HCl buffer (20 mM, pH 7.4) contained 1 mM phenylmethylsulfonyl fluoride (PMSF), 1 mM sodium orthovanadate, 1 mM sodium fluoride, 1 mM EDTA, 0.32 M sucrose and a complete protease inhibitor cocktail were used for homogenizing tissue. Then, the supernatants were obtained from the homogenates after centrifugation at 14,000 rpm for 20 min at 4 °C. The 15 µg of protein were detected using BCA assay kit and were separated on 10% sodium dodecyl sulfate polyacrylamide gel electrophoresis (SDS-PAGE) gels under reducing conditions. The proteins were blotted on polyvinylidene difluoride (PVDF) membranes in Tris–HCl transfer buffer (25 mM, pH 7.4) containing 192 mM glycine and 20% methanol (v/v) at 300 mA for 2 h. The membranes were blocked with 5% skim milk for 2 h at room temperature and then incubated with primary antibodies at 4 °C overnight. Then, the PVDF membranes were washed and incubated with a suitable secondary antibody for 2 h at room temperature. And then the membranes were incubated with enhanced chemiluminescence reagent (Amersham™ ECL Select™ Western Blotting Detection Reagent or Thermo Scientific SuperSignal™ West Pico PLUS Chemiluminescent Substrates). Protein signals were imaged using the Molecular Imager® ChemiDoc™ XRS System with İmage Lab™ Software (Bio-Rad Laboratory, Berkeley, CA) and analyzed using the Image J analysis program, as described elsewhere [30].

### Statistical analysis

Statistical analyses were conducted using Prism 7.0 software (GraphPad, La Jolla, CA). The results are presented as the means ± standard errors of the means (SEM). The results were analyzed by one-way analysis of variance (ANOVA), and multiple comparisons were performed using the Student–Newman–Keuls test. Two-way ANOVA followed by Bonferroni’s post hoc test was performed for the object preference ratio during the novel object recognition test, PPI (%) and startle amplitude in the ASR test. Differences were considered significant at the level of *P* < 0.05. Animal behaviors were analyzed by researchers who had no information about the treatments administered. The group sizes and statistical analyses for behavioral tests and western blot analyses are provided in the Supplementary Table S1.

## Results

### Total phenolic content and radical scavenging effect of C. myxa leaves

Total phenolic contents and radical scavenging effects were determined *in vitro* using conventional methods. The total phenolic content was determined to be 401.09 ± 41.47 mg GAE/g of dry weight of the *C. myxa* leaf extract. The extract showed a moderate DPPH radical scavenging effect with an IC_50_ of 336.58 ± 19.37 µg/ml.

### Quantification of rosmarinic acid in ELCM by HPLC analysis

**Fig. 1:**
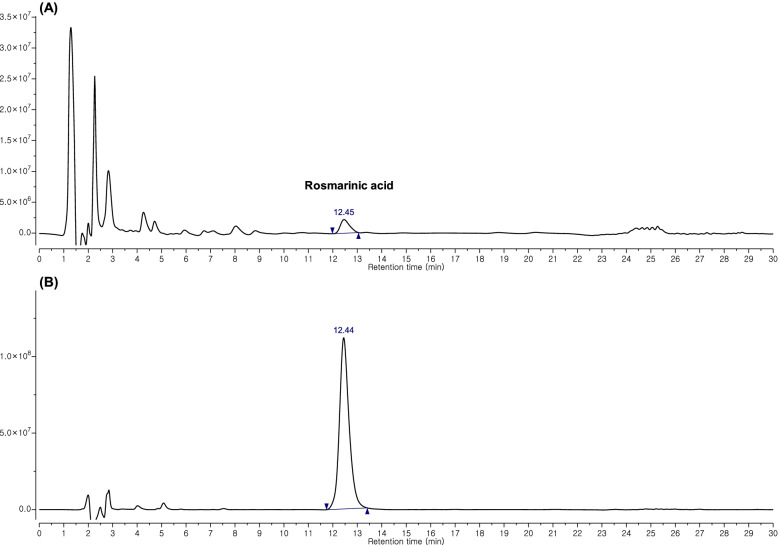
Analytical chromatograms of the 70% EtOH extract of the *C. myxa* leaf extract (ELCM) (**A**) and rosmarinic acid (**B**) were acquired at 330 nm. The retention time of rosmarinic acid was 12.44 min. The concentration of rosmarinic acid in ELCM was 4.66 ± 0.02 mg/g

In comparison to the retention time (Rt) and photodiode array spectra of the reference solution, the HPLC chromatograms also revealed the presence of rosmarinic acid (Rt = 12.44 min) in ELCM. The calibration curve of the standard was obtained with serially diluted solutions (62.5–1000 mg/ml) in order to quantify rosmarinic acid in ELCM. The regression equation was y = 267.310x – 805.042 (*r*2 = 0.999, *n* = 5). An average content of rosmarinic acid in ELCM was determined to be 4.66 ± 0.02 mg/g (Fig. 1). Rutin was also identified as one of the major compounds in ELCM by an UPLC-MS analysis (Supplementary Fig. S1).

### The effect of ELCM on long-term memory in the passive avoidance task

**Fig. 2:**
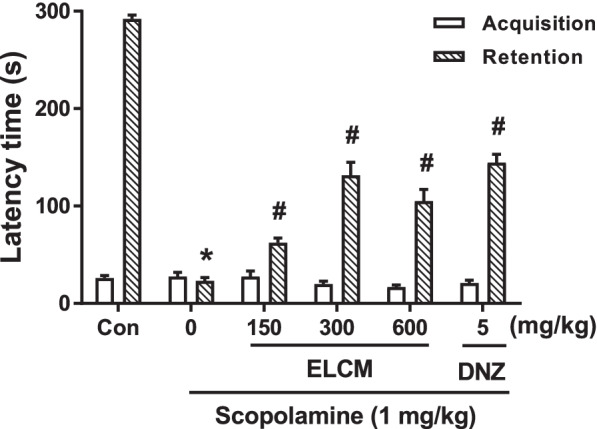
ELCM ameliorated scopolamine-induced memory deficits in the passive avoidance test. ELCM (150, 300 and 600 mg/kg), donepezil (DNZ, 5 mg/kg) and scopolamine (1 mg/kg) were administered to mice. The results from the acquisition trial and retention trial are presented. Data are presented as the means ± S.E.M. (*n* = 10/group) (*P < 0.05 compared with the naïve control group; #*P* < 0.05 compared with the scopolamine-treated group). ELCM, 70% ethanol extract of *C. myxa* leaves; Con, control; DNZ, donepezil

The passive avoidance test, which is mostly dependent on long-term memory, was performed to evaluate the effect of ELCM on ameliorating scopolamine-induced memory impairment. Significant differences were not observed in the latency times during the acquisition trial among all groups (Fig. 2). Significant group effects on the latency times during the retention trial were observed. The scopolamine-induced reduction in latency time was significantly reversed in mice treated with ELCM (150, 300 and 600 mg/kg) and donepezil (*P* < 0.05).

### The effect of ELCM on performance in the novel object recognition task

**Fig. 3:**
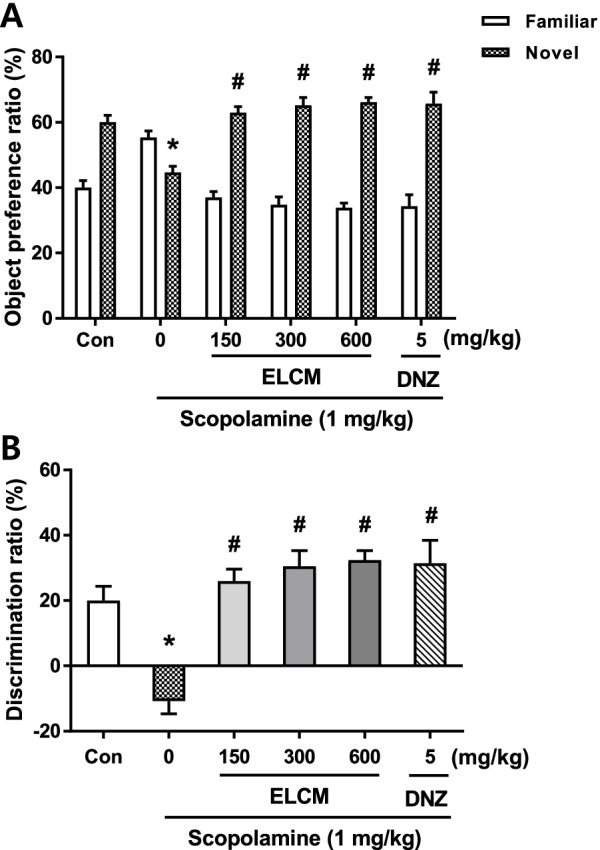
ELCM ameliorated scopolamine-induced memory deficits in the novel object recognition test. ELCM (150, 300 and 600 mg/kg), donepezil (DNZ, 5 mg/kg) and scopolamine (1 mg/kg) were administered to mice. The results for the object preference ratio (**A**) and discrimination ratio (**B**) are provided. Data are presented as the means ± S.E.M. (*n* = 9–10/group) (*P < 0.05 compared with the naïve control group; #*P* < 0.05 compared with the scopolamine-treated group). ELCM, 70% ethanol extract of *C. myxa* leaves; Con, control; DNZ, donepezil

The novel object recognition test was performed to evaluate the effect of ELCM on object recognition memory in mice. There were significant group effect in both the object preference ratio (Fig. 3A) and the discrimination ratio (Fig. 3B). A reduced preference for the novel object and a decreased discrimination ratio between the novel and familiar objects were observed in mice treated with scopolamine. The administration of ELCM (150, 300 and 600 mg/kg, p.o.) and donepezil significantly attenuated the scopolamine-induced cognitive impairment.

### The effect of ELCM on the startle amplitude and PPI in the ASR test

**Fig. 4:**
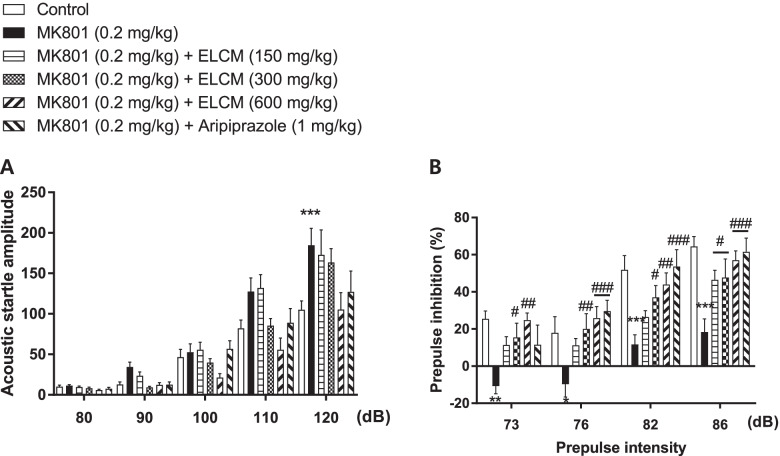
The effects of ELCM on the acoustic startle response and prepulse inhibition (%) in the acoustic startle response task. ELCM (150, 300 and 600 mg/kg p.o.), aripiprazole (1 mg/kg, i.p.) and MK-801 (0.2 mg/kg, i.p.) were administered to mice. The acoustic startle response (**A**) and the percentage of prepulse inhibition (**B**) with ELCM treatment in the MK-801-induced PPI deficits were presented. Data are presented as the means ± S.E.M. (*n* = 9–10/group) (*P < 0.05 compared with the naïve control group; #*P* < 0.05 compared with the MK-801-treated group). ELCM, 70% ethanol extract of *C. myxa* leaves

We conducted the ASR test to investigate the effect of ELCM on startle amplitude and PPI impairment in MK-801-induced mice. There were significant group effects on the startle amplitude (Fig. 4A) and PPI (Fig. 4B) related to treatment and pulse intensity in the ASR test. The administration of MK-801 (0.2 mg/kg, i.p.) significantly increased the startle amplitude at the 120 dB pulse level for acoustic startle amplitude. It decreased the PPI at all prepulse levels compared with the naïve control group (*P* < 0.05). Although the ELCM was not attenuated the startle amplitude on MK-801-induced hypersensitivity in mice (Fig. 4A), the disrupted PPI was ameliorated by the treatment with ELCM (150, 300, or 600 mg/kg) at all prepulse levels compared with the MK-801-treated group (*P* < 0.05, Fig. 4B). In addition, the positive control, Aripiprazole, significantly ameliorated the MK-801-induced increases in startle responses and PPI disruptions (*P* < 0.05).

### The effect of ELCM on memory-related signaling pathways in the cortex and hippocampus

**Fig. 5:**
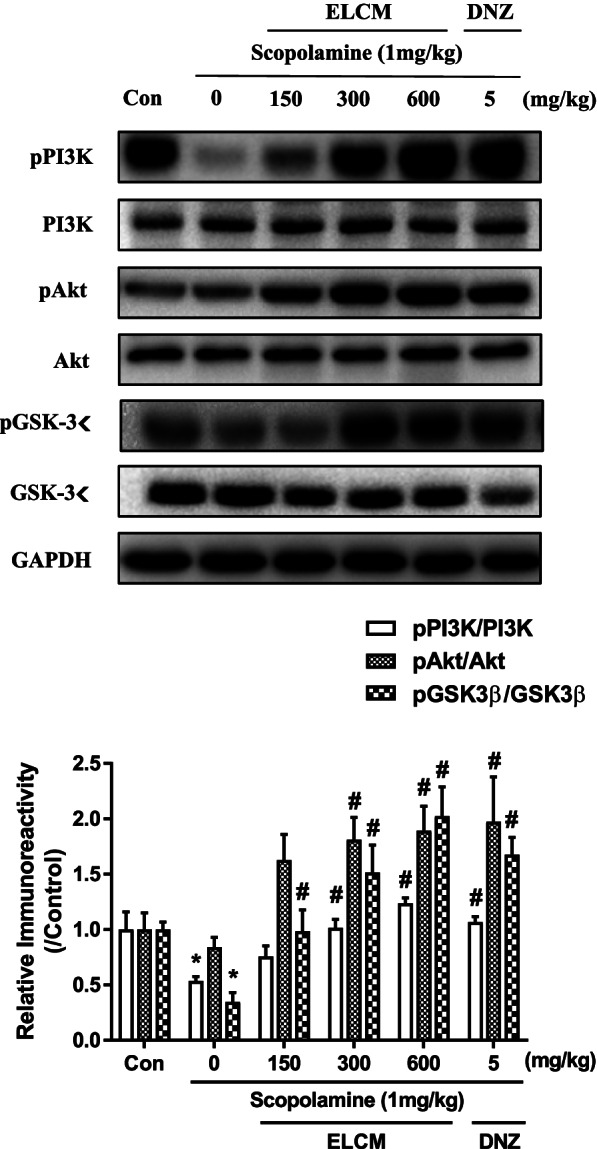
The phosphorylation levels of PI3K, Akt and GSK-3β in the cortex after the administration of ELCM. Mice were sacrificed 1 h after the administration of ELCM (150, 300 or 600 mg/kg, p.o.) and donepezil (DNZ, 5 mg/kg, p.o.). The immunoreactivity of PI3K, Akt, and GSK-3β and their phosphorylation levels in the cortical tissues were measured. The immunoreactivity levels of pPI3K/PI3K, pAkt/Akt and pGSK-3β/GSK-3β were standardized to those in the control group (set to 1.0). Data are presented as the means ± S.E.M. (*n* = 6/group) (*P < 0.05 compared with the naïve control group; #*P* < 0.05 compared with the scopolamine-treated group). ELCM, 70% ethanol extract of *C. myxa* leaves; Con, control; DNZ, donepezil

We performed Western blotting to determine which signaling molecule(s) involved in cognitive function were affected by ELCM. One-way ANOVA revealed significant group effects on the phosphorylation levels of PI3K, Akt and GSK-3β in the cortex. ELCM (300 or 600 mg/kg, p.o.) significantly reversed the decreases in levels of PI3K, Akt and GSK-3β phosphorylation induced by scopolamine treatment (*P* < 0.05, Fig. 5).

**Fig. 6:**
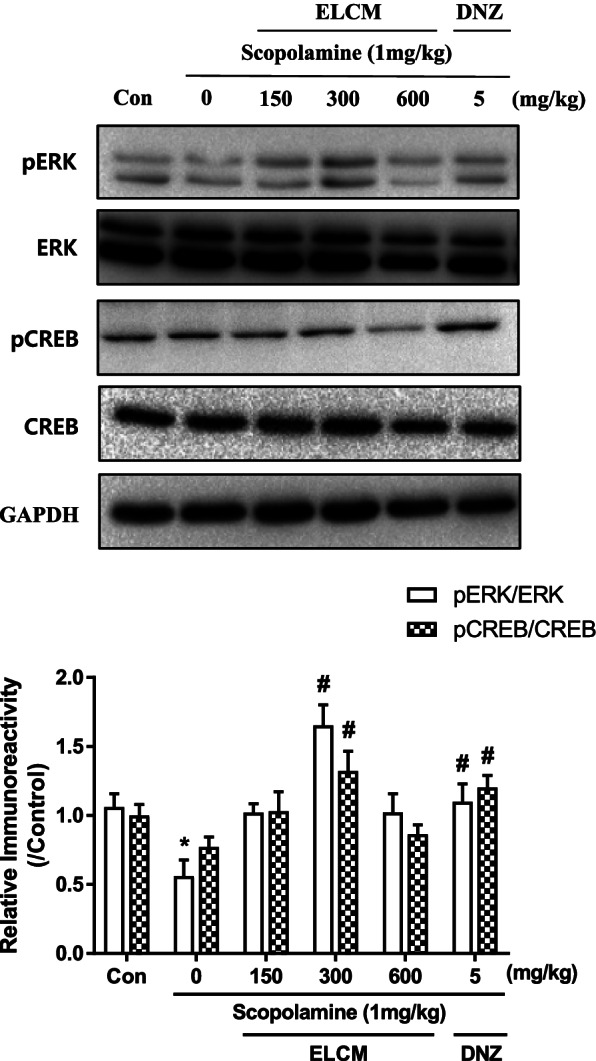
The phosphorylation levels of ERK and CREB in the hippocampus after the ELCM administration. Mice were sacrificed 1 h after the administration of ELCM (150, 300 or 600 mg/kg, p.o.) and donepezil (DNZ, 5 mg/kg, p.o.). The immunoreactivity of ERK and CREB and their phosphorylation levels in the hippocampal tissues were measured. The immunoreactivity levels of pERK/ERK and pCREB/CREB were standardized to those in the control group (set to 1.0). Data are presented as the means ± S.E.M. (*n* = 6/group) (*P < 0.05 compared with the naïve control group; #*P* < 0.05 compared with the scopolamine-treated group). ELCM, 70% ethanol extract of *C. myxa* leaves; Con, control; DNZ, donepezil

On the other hand, the levels of phosphorylated ERK and CREB in the hippocampus showed group differences. The levels of pERK and pCREB were significantly increased in the groups administered of ELCM (300 mg/kg, p.o.) compared to the scopolamine-treated group (*P* < 0.05, Fig. 6). However, the phosphorylation levels of Akt and GSK-3β in the hippocampus were slightly increased upon the administration of ELCM, but the differences were not statistically significant (data not shown).

## Discussion

In the present study, we found that ELCM ameliorated scopolamine-induced cognitive impairment in mice measured by the passive avoidance task and the novel objective recognition tasks and reversed MK-801-induced deficits in sensorimotor gating functions. Western blot analysis revealed that ELCM increased the levels of signaling molecules related to learning and memory behaviors, such as levels of phosphorylated PI3K, Akt and GSK-3β in the cortex and levels of phosphorylated ERK and CREB in the hippocampus.

It is well known that recognition memory deficits are an early symptom observed in patients with AD [31, 32]. Even mild AD patients also exhibit impairments in several stages of object recognition processes, in part, due to problems in retrieval from long-term semantic memory [32]. In the present study, we employed the novel object recognition task to evaluate object recognition memory. Object recognition is measured by the difference in the time spent exploring novel or familiar objects. ELCM administration exerted a positive effect on performance in the novel object recognition task, ameliorating the scopolamine-induced decreased in the discrimination ratio and preference for the novel object. Similarly, ELCM also significantly reversed the scopolamine-induced reduction in latency to enter the dark box in the passive avoidance task, which is a fear-motivated test related to long-term memory [33]. Based on these results, ELCM ameliorates the cognitive impairment in mice with cholinergic dysfunction. ELCM contains phenolic components and possesses antioxidant properties. The level of total phenolic content in ELCM was 401.09 mg GAE/g, which was relatively higher than those in the fruit extract of *C. myxa* (98.84 ± 0.50 mg GAE/g) [34]. Accordingly, the antioxidant activity of ELCM was also higher than that of *C. myxa* fruit extract (ELCM, 336.58 ± 19.37 mg/ml; *C. myxa* fruit extract, IC_50_ > 6,000 mg/ml) [34]. Recently, it has been reported that rutin is contained together with *p*-coumaric acid or caffeic acid in the *C. myxa* leaves extract [35]. We further identified the presence of rutin together with rosmarinic acid in ELCM (supplementary Fig. S1). Rosmarinic acid is contained in ELCM at a relatively high concentration (4.66 ± 0.02 mg/g) and might be expected to exert the representative activity of ELCM because it displays various biological activities, such as antioxidant, anti-inflammatory and neuroprotective effects [36–38]. Furthermore, rosmarinic acid has been reported as an AChE inhibitor with a very low Ki value [39], suggesting that rosmarinic acid would be a major active compound in ELCM responsible for ameliorating cognitive dysfunction. Therefore, we attempted to investigate whether ELCM inhibits AChE activity. However, we did not observe any inhibitory activity on AChE in an *in vitro* study (data not shown), suggesting that cognitive dysfunction ameliorating activities of ELCM might not be derived from its direct inhibition of AChE activity. Further studies are needed to clarify these issues.

Patients with early AD show deficits in sensorimotor gating function [40]. Sensorimotor gating is a process that filters environmental stimuli, resulting in appropriate responses to the demands of the environment. As a measure of sensorimotor gating, PPI of the startle response is impaired in patients with some neuropsychiatric disorders [41, 42]. Since PPI impairments have been observed in individuals with early AD, PPI has been suggested as a biomarker for the clinical diagnosis and detection of the risk of AD [43]. In addition, the hippocampus and entorhinal cortex, which are affected in patients with mild AD, play roles in regulating prepulse inhibition [44]. Therefore, we investigated whether ELCM may reverse the impaired PPI induced by NMDA receptor blockade. MK-801-induced PPI deficits were significantly ameliorated by ELCM treatments. Thus, ELCM may ameliorate the disruption of sensorimotor gating observed in patients with AD.

It has been shown that the PI3K-Akt-GSK-3β and ERK-CREB signaling pathways play roles in neuroprotection and enhancing cell survival by stimulating cell proliferation and inhibiting apoptosis [45]. In addition, the ERK-CREB signaling pathway plays crucial roles in memory acquisition and retrieval by regulating the expression of nerve growth factors, especially in the hippocampus [46]. These signaling pathways also appear to be important in the progression of AD because of their association with Tau protein hyperphosphorylation by GSK-3β and CREB [47, 48]. In the present study, we found that the administration of ELCM increased the phosphorylation levels of PI3K, Akt and GSK-3β in the cortex and the phosphorylation levels of ERK and CREB in the hippocampus. These findings suggest that ELCM could decreased the phosphorylation levels of Tau protein. And the cognitive ameliorating effects of ELCM would be derived by these signaling pathway enhancements. However, the levels of pERK and pCREB at 600 mg/kg of ELCM were lower than those at 300 mg/kg of ELCM. We did not have any clue to explain this issue. It can be speculated that neurotransmitters released by higher dose of ELCM could activate autoreceptors, resulting in the inhibition of the release of relevant neurotransmitters and inhibition of neuronal activities, such as decreased phosphorylation levels of signaling molecules [49]. Similar inverted U-shaped dose–response patterns have been reported by us and others [50–52]. Further studies are needed to clarify this issue. Additionally, the Akt-GSK-3β signaling pathway plays an important role in the sensorimotor gating function, which is obviously characterized by PPI [53]. Regulating levels of phosphorylated Akt and GSK-3β attenuats cognitive dysfunction and PPI deficit in MK-801-treated mice [54, 55]. These findings suggested that the effect of ELCM on ameliorating the PPI deficit might be related to the increased levels of phosphorylated Akt and GSK-3β in the cortex.

In the tropical regions, the leaves of *C. myxa* has been used for treating *T. cruzi* infection-induced trypanosomiasis [20]. *T. cruzi* infection is treated with some anti-parasitic agents, such as benznidazole, for acute infection [56]. It has been reported that chronic infections (30 to 40 years) with *T. cruzi* affect cognitive impairment [57]. To date, however, there were no studies on the effect of *C. myxa* against *T. cruzi* infection-induced cognitive impairment in vitro or in vivo. Therefore, our present results support ELCM as a potential treatment for *T. cruzi* infection-induced cognitive impairment although we did not investigate the effect of ELCM against trypanosomiasis or cognitive dysfunction caused by *T. cruzi* infection.

In conclusion, the administration of ELCM significantly ameliorates scopolamine-induced memory impairments and MK-801-induced sensorimotor gating deficits. These behavioral outcomes would be mediated by the activation of the PI3K-Akt-GSK-3β signaling pathway in the cortex and the ERK-CREB signaling pathway in the hippocampus, which are related to learning and memory. Based on these results, ECLM would be a potential therapeutic agent for cognitive dysfunction and sensorimotor gating disruptions observed in individuals with neurodegenerative diseases, including AD.

## Supplementary Information

**Additional file 1. **Supplementarymaterials and methods.

**Additional file 2: Table S1.** Statistical analysis of figures.

**Additional file 3: Figure S1.**UPLC-MS fingerprint analysis data of ELCM.

**Additional file 4.**

## Data Availability

The datasets used and analyzed during the current study are available from the corresponding author on reasonable request.

## Abbreviations

Aβ: Amyloid-β
AChE: Acetylcholinesterase
ANOVA: Analysis of variance
BCA: Bicinchoninic acid
Akt: Protein kinase B
GSK3β: Glycogen synthase kinase 3β
PI3K: Phosphoinositide 3-kinase
ERK: Extracellular signal-regulated kinase
CREB: CAMP response element-binding protein
GAPDH: Glyceraldehyde 3-phosphate dehydrogenase
PVDF: Polyvinylidene difluoride
