# Supplementary material for: The effects of the ethanol extract of Cordia myxa leaves on the cognitive function in mice
Source: BMC Complement Med Ther. 2022 Aug 10;22:215. doi: 10.1186/s12906-022-03693-z (PMC9367120; doi:10.1186/s12906-022-03693-z)
Supplement: Supplementary file 1 — Additional file 1. Supplementarymaterials and methods. [file 12906_2022_3693_MOESM1_ESM.docx]

Supplementary Materials and methods

*Materials for antibodies*

Mouse monoclonal anti-protein kinase B (Akt), rabbit polyclonal anti-phosphorylated Akt (pAkt), mouse monoclonal anti-glycogen synthase kinase (GSK)-3β, mouse monoclonal anti-extracellular signal-regulated kinase (ERK), mouse monoclonal anti-cAMP response element-binding protein (CREB) and mouse monoclonal anti-glyceraldehyde 3-phosphate dehydrogenase (GAPDH) antibodies were procured from Santa Cruz Biotechnology, Inc. (Santa Cruz, CA). Rabbit polyclonal anti-phosphoinositide 3-kinase (PI3K), rabbit polyclonal anti-phosphorylated PI3K (pPI3K), rabbit polyclonal anti-phosphorylated ERK (pERK), rabbit monoclonal anti-phosphorylated CREB (pCREB) and mouse monoclonal anti-phosphorylated GSK-3β antibodies were obtained from Cell Signaling Technology (Danvers, MA). Horseradish peroxidase-conjugated anti-rabbit and anti-mouse secondary antibodies were obtained from GeneTex Inc. (Irvine, CA).
