# Supplementary material for: The effects of the ethanol extract of Cordia myxa leaves on the cognitive function in mice
Source: BMC Complement Med Ther. 2022 Aug 10;22:215. doi: 10.1186/s12906-022-03693-z (PMC9367120; doi:10.1186/s12906-022-03693-z)
Supplement: Supplementary file 3 — Additional file 3: Figure S1.UPLC-MS fingerprint analysis data of ELCM. [file 12906_2022_3693_MOESM3_ESM.docx]

Supplementary information

**The effects of the ethanol extract of *Cordia myxa* leaves on the cognitive function in mice**

**Contents**

**Figure S1.** UPLC-MS fingerprint analysis data of ELCM

**Figure S1.** UPLC-MS fingerprint analysis data of ELCM

**
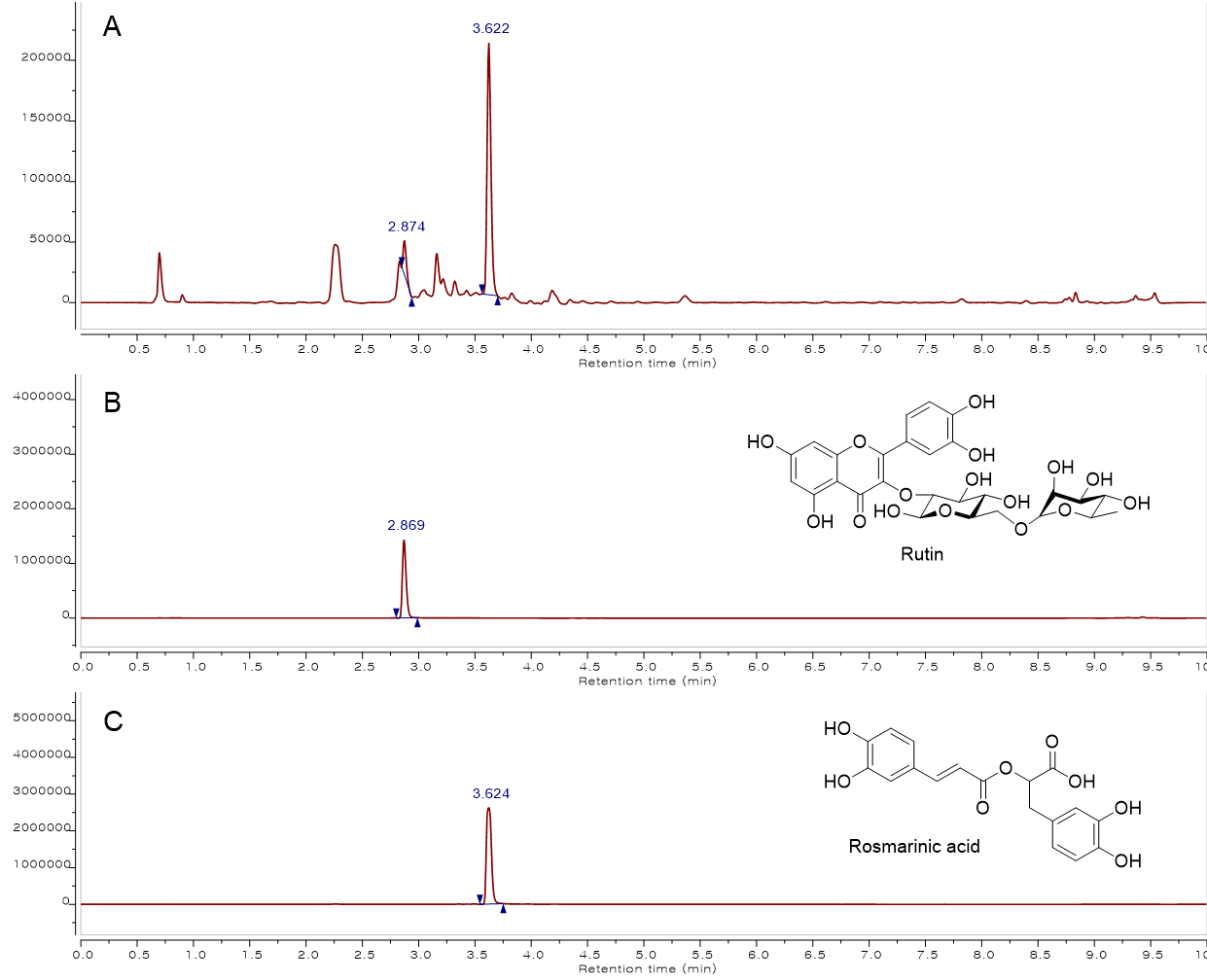
**

UPLC chromatogram of ELCM (A), rutin (B; *R_t_*: 2.869 min), and rosmarinic acid (C; *R_t_*: 3.624 min) at 330 nm. UPLC chromatogram were collected by Waters Aquity UPLC system.
