# Supplementary material for: The effects of the ethanol extract of Cordia myxa leaves on the cognitive function in mice
Source: BMC Complement Med Ther. 2022 Aug 10;22:215. doi: 10.1186/s12906-022-03693-z (PMC9367120; doi:10.1186/s12906-022-03693-z)
Supplement: Supplementary file 4 — Additional file 4. [file 12906_2022_3693_MOESM4_ESM.docx]

Supplementary information

**The effects of the ethanol extract of *Cordia myxa* leaves on the cognitive function in mice**

**Author explanation:**

We guarantee that the given western blot was cropped from the same full-length blot. We only crop the captured image to present the region of interest.

Here we have presented the Original images of all blots.


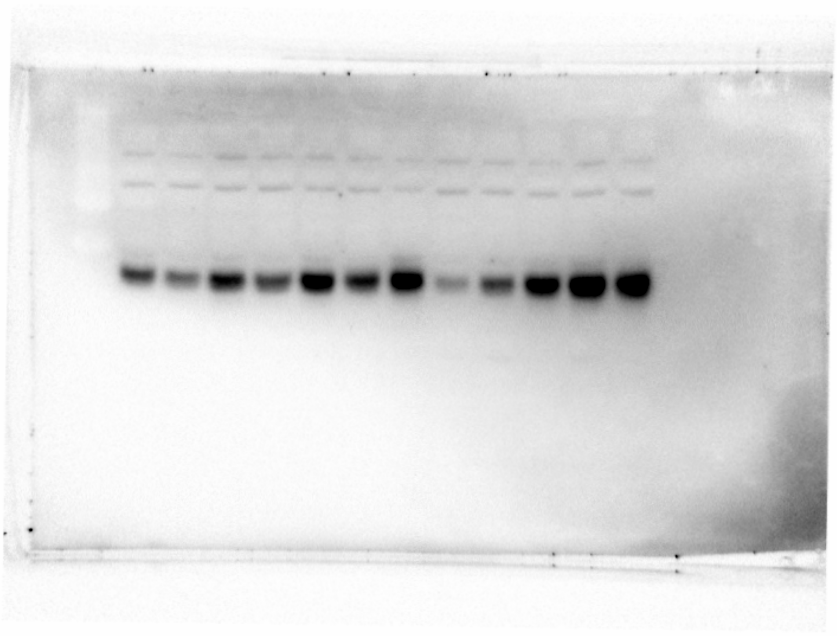

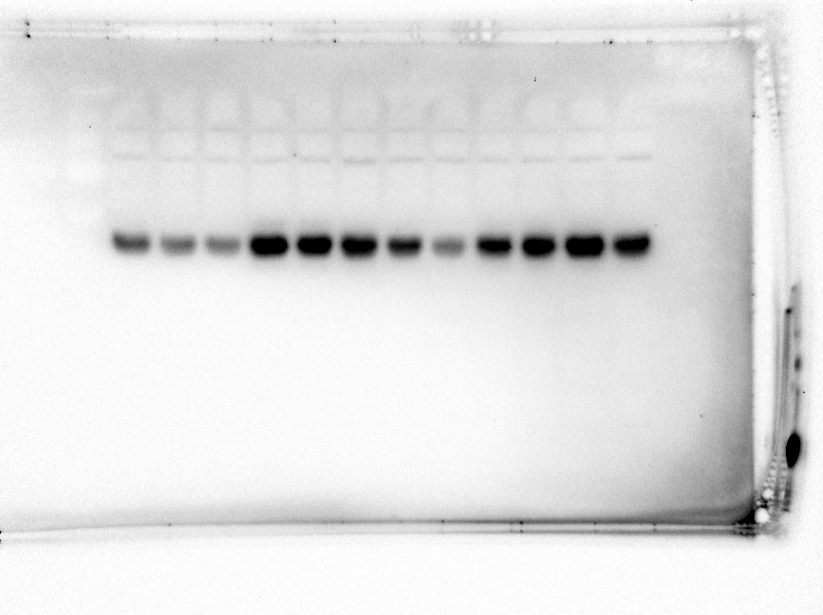

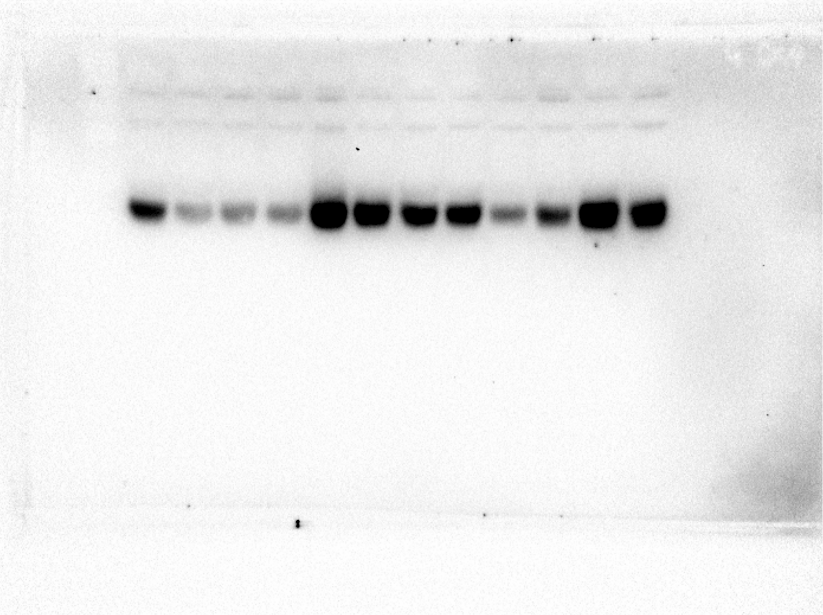


Fig. 5; pPI3K


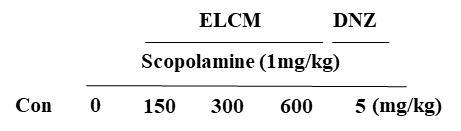

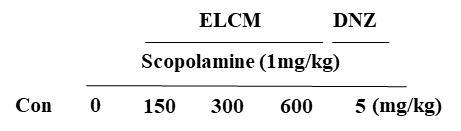

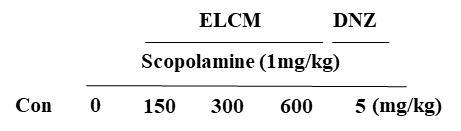

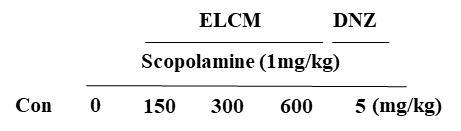

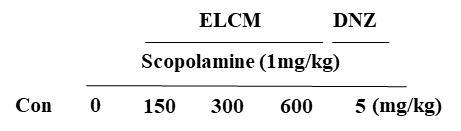

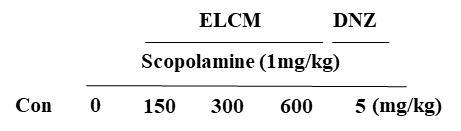


p-PI3K
(60 kDa)

p-PI3K
(60 kDa)

p-PI3K
(60 kDa)


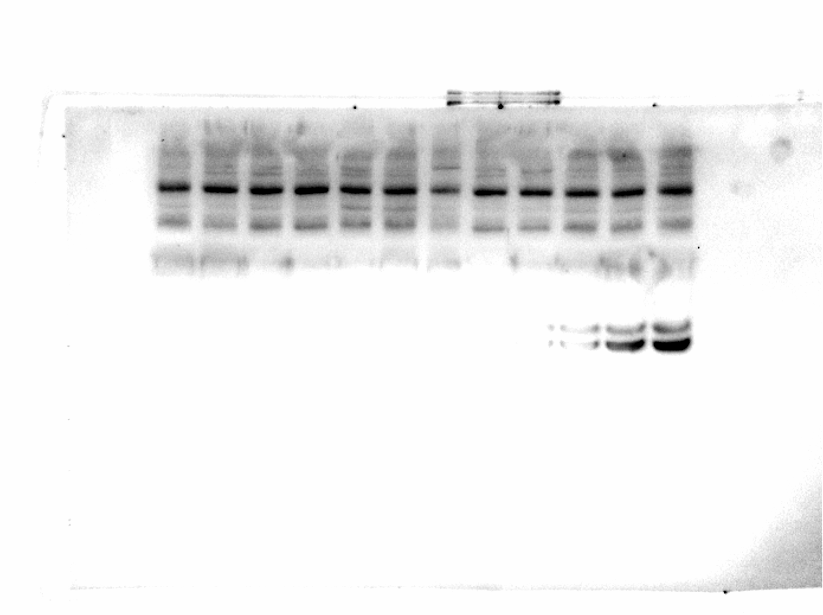

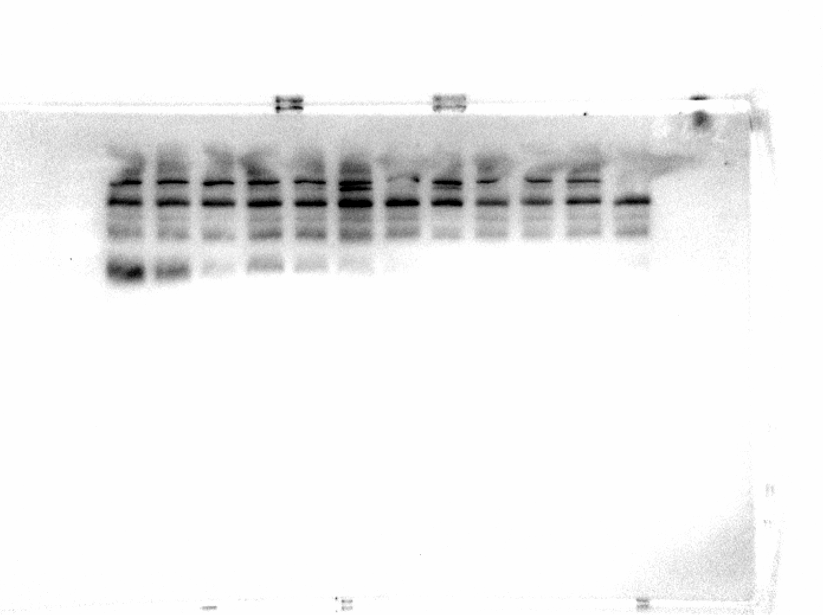

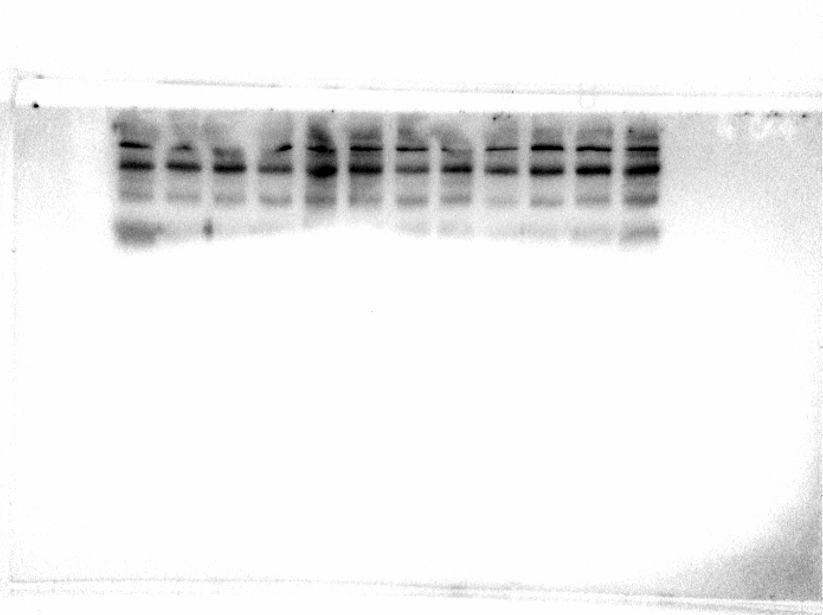


Fig. 5; t-PI3K

PI3K
(80 kDa)

PI3K
(80 kDa)

PI3K
(80 kDa)


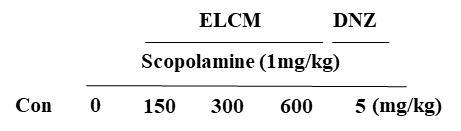

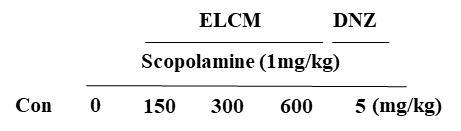

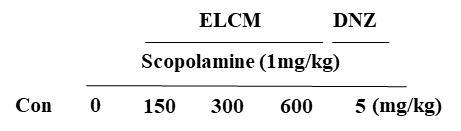

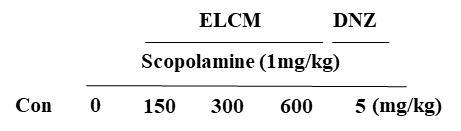

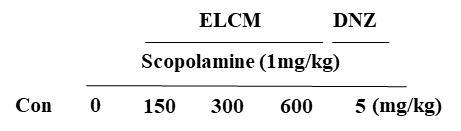

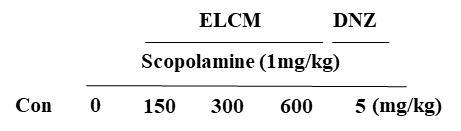


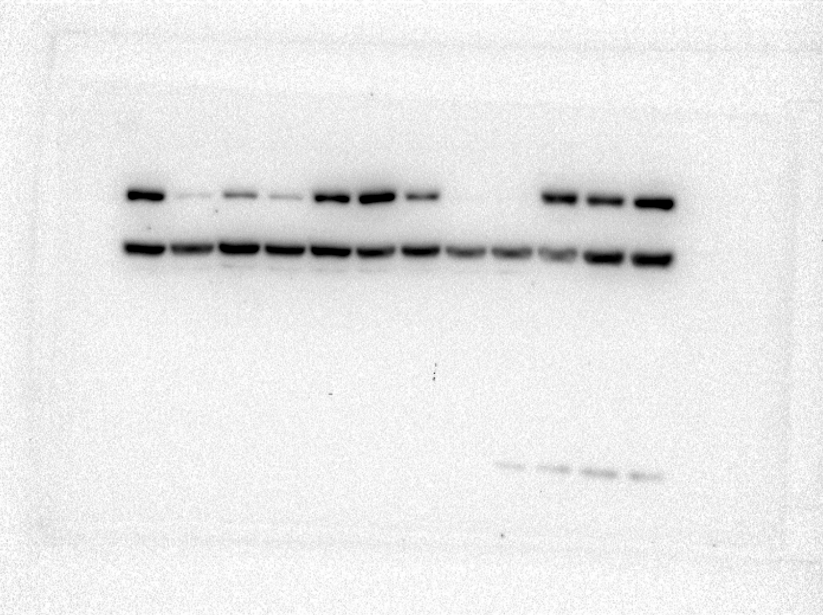

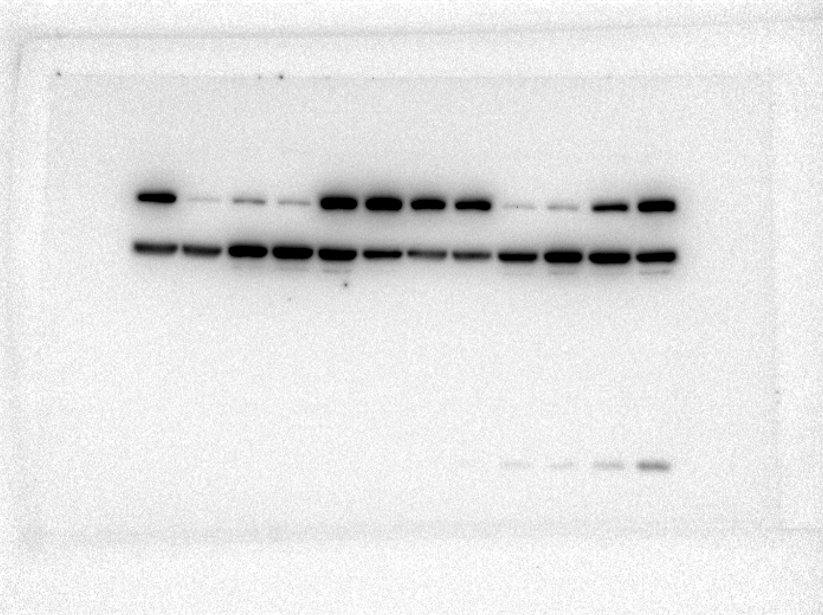

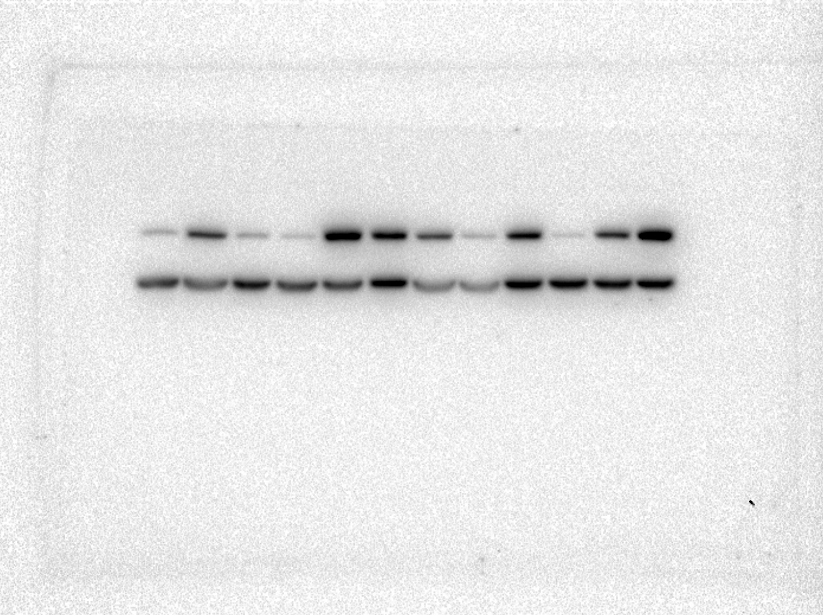


Fig. 5; p-Akt

p-Akt

(60 Kda)

p-Akt

(60 Kda)

p-Akt

(60 Kda)


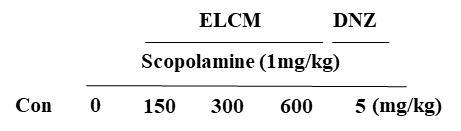

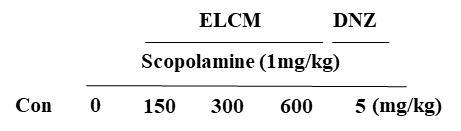

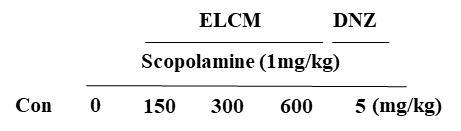

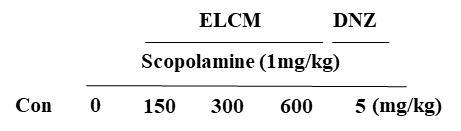

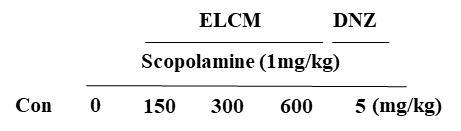

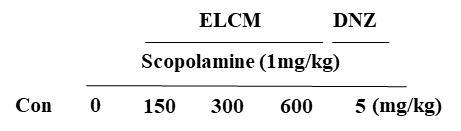


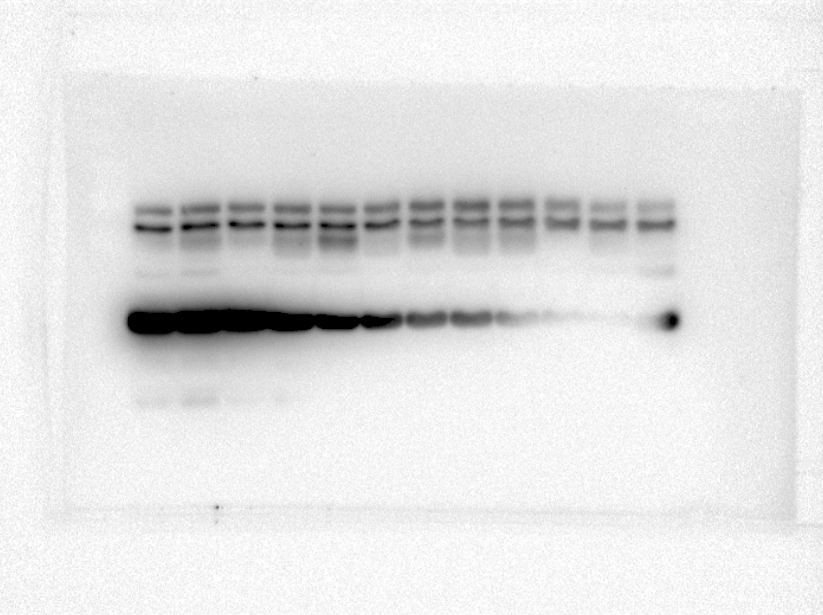

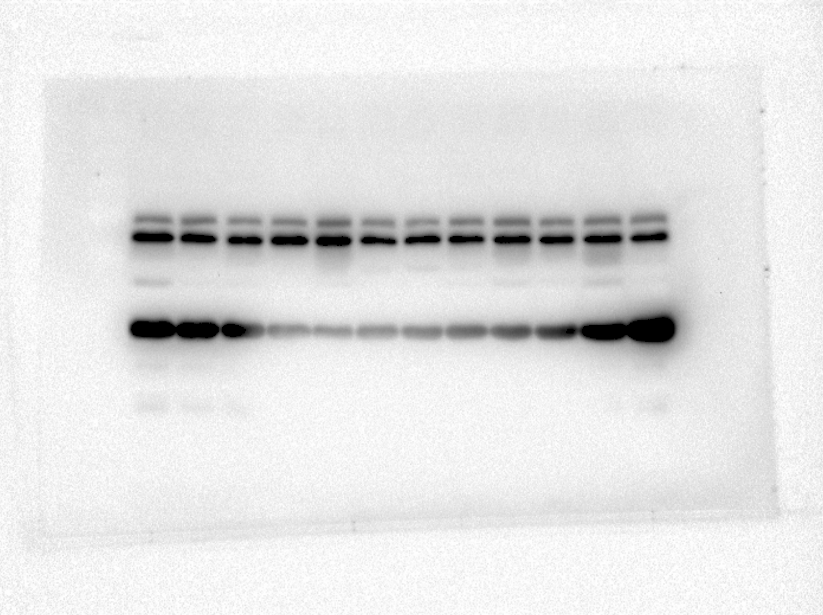

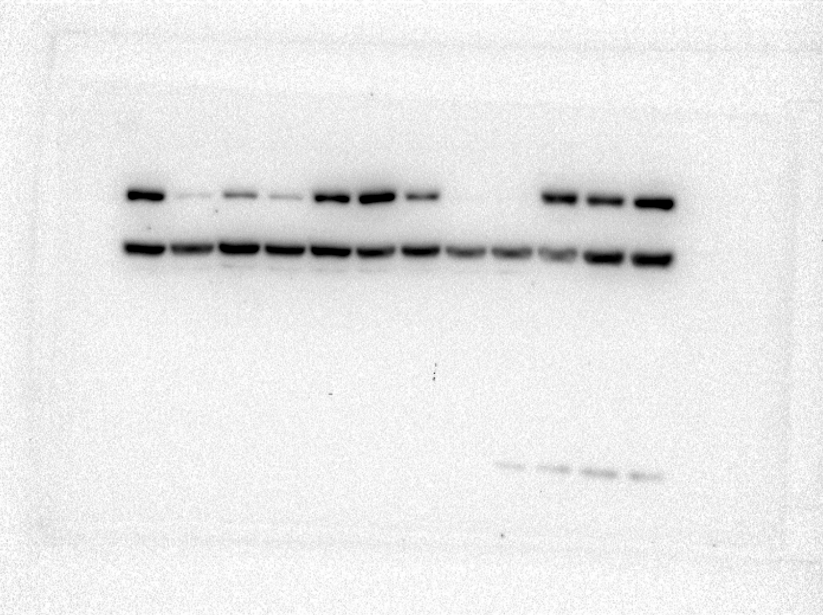


Fig. 5; t-Akt

t-Akt

(60 Kda)

t-Akt

(60 Kda)

t-Akt

(60 Kda)


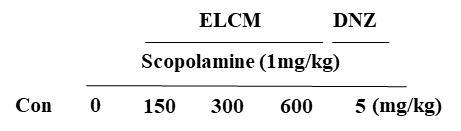

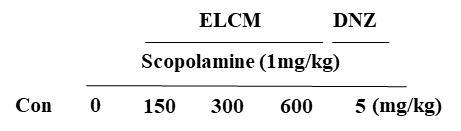

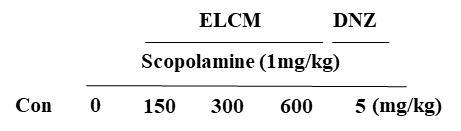

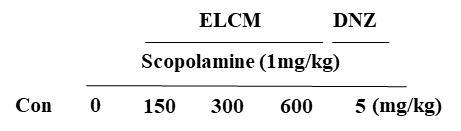

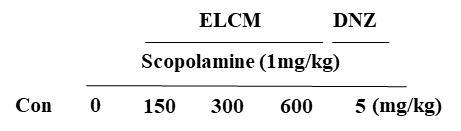

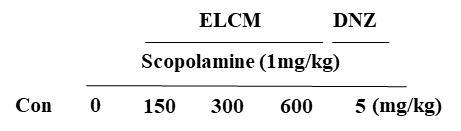


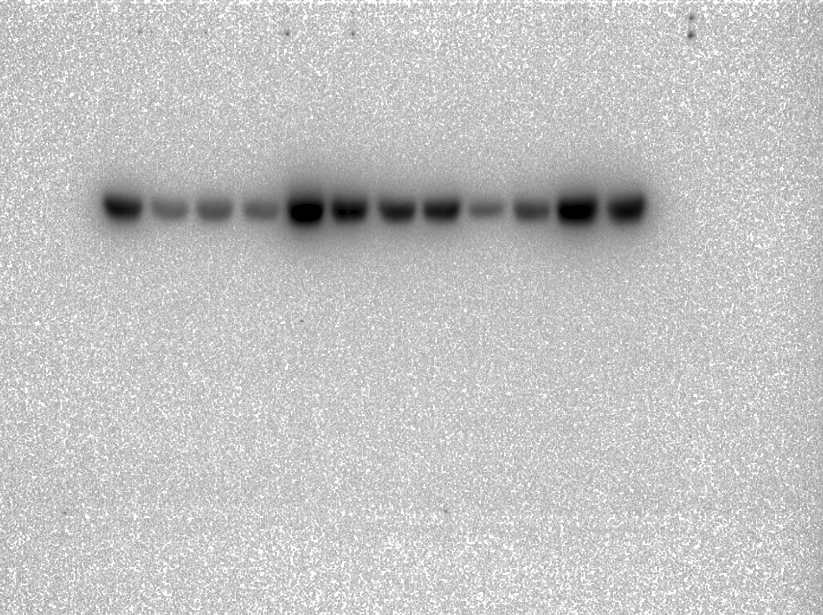

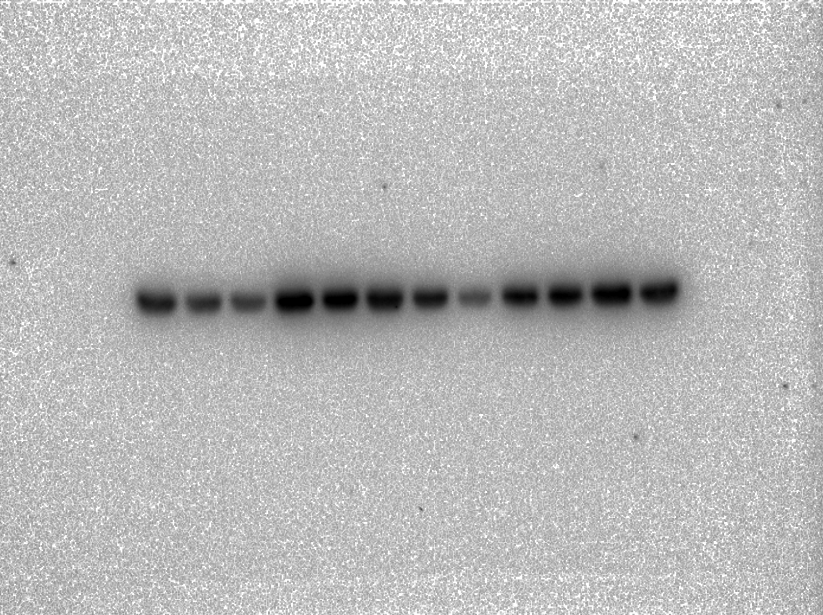

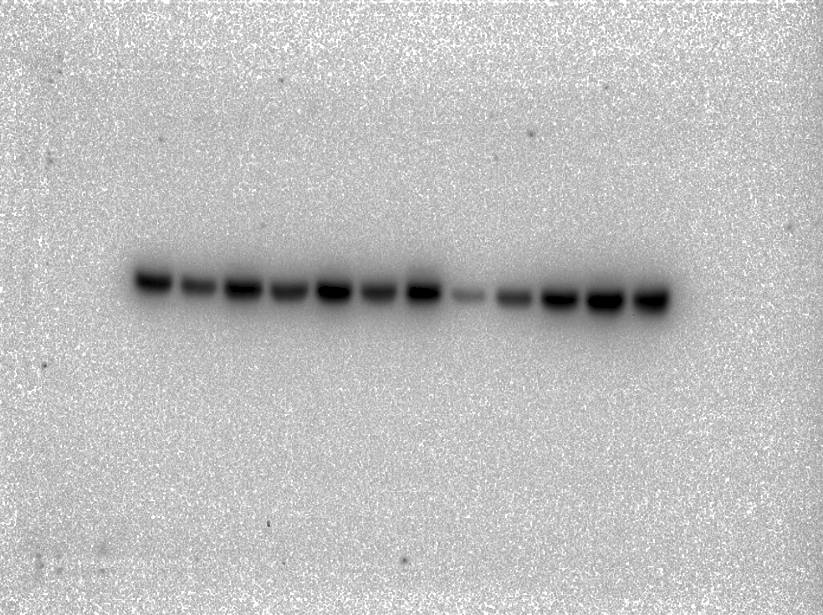


Fig. 5; p-GSK3β

p-GSK3β

(46 Kda)

p-GSK3β

(46 Kda)

p-GSK3β

(46 Kda)


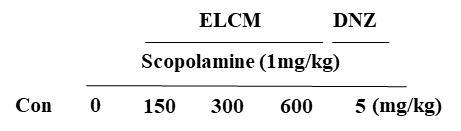

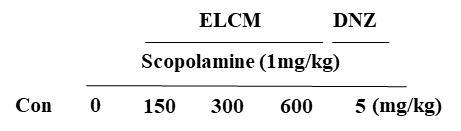

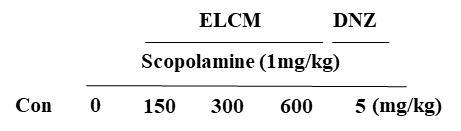

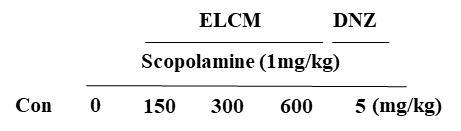

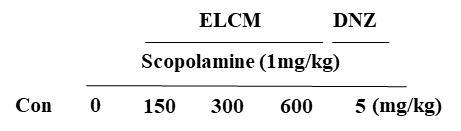

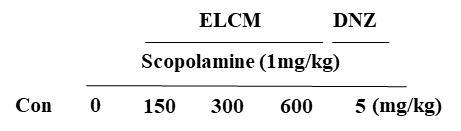

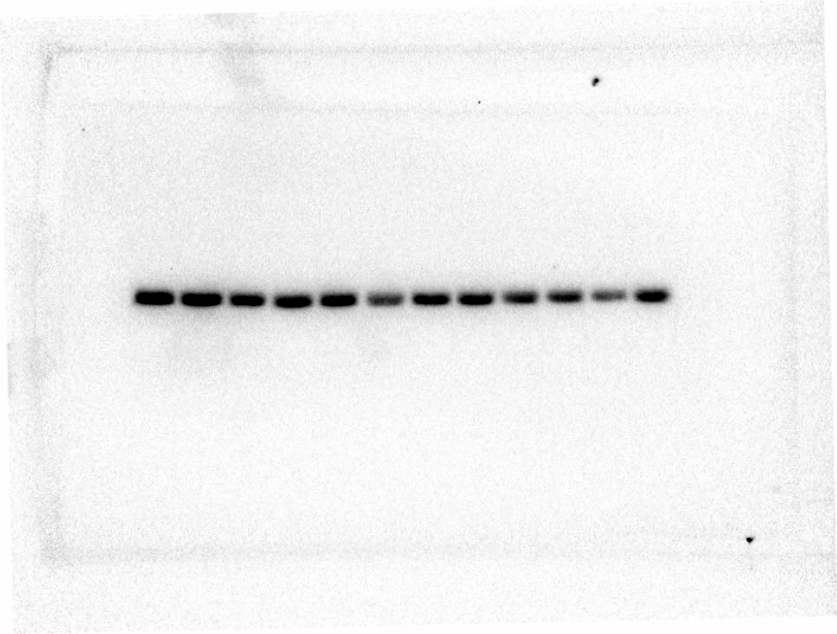

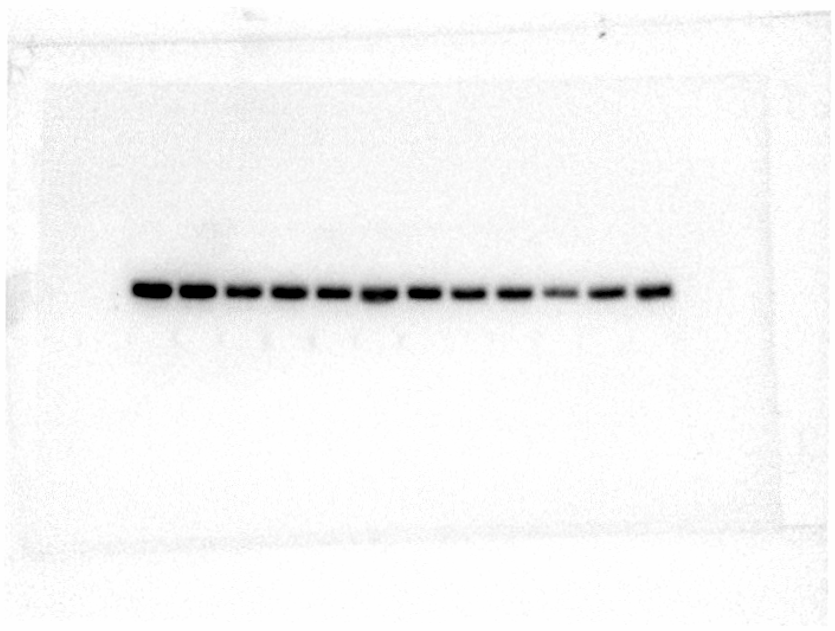

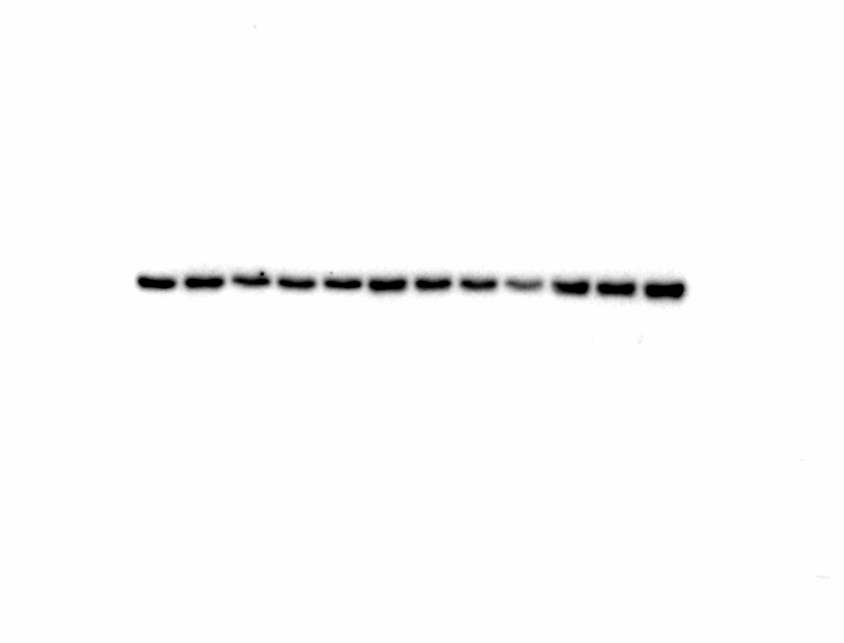


Fig. 5; t-GSK3β

t-GSK3β

(46 Kda)

t-GSK3β

(46 Kda)

t-GSK3β

(46 Kda)


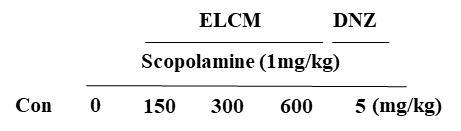

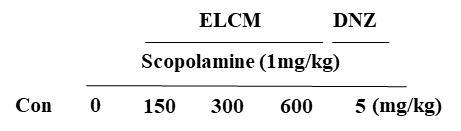

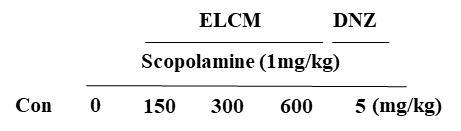

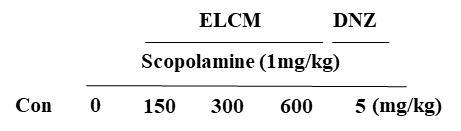

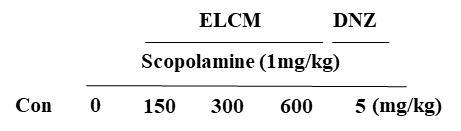

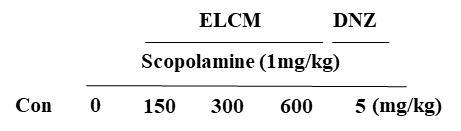


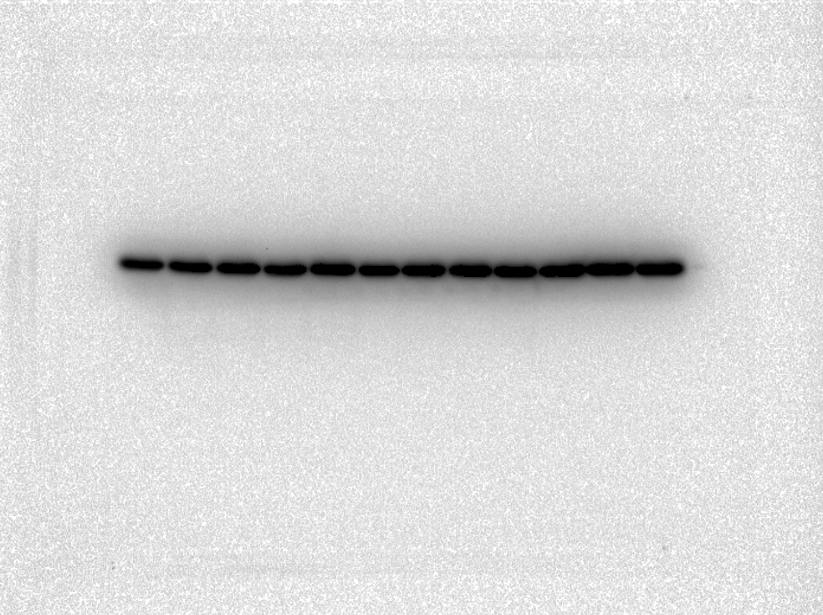

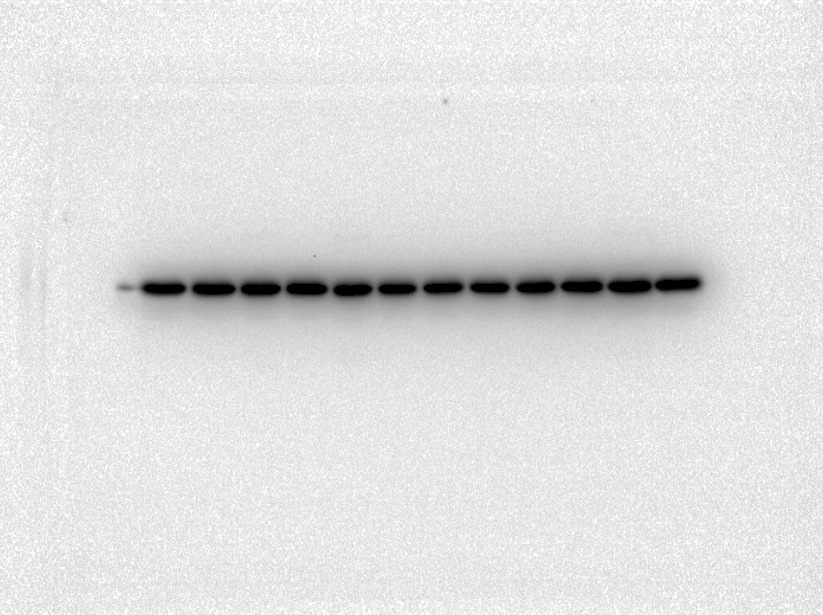

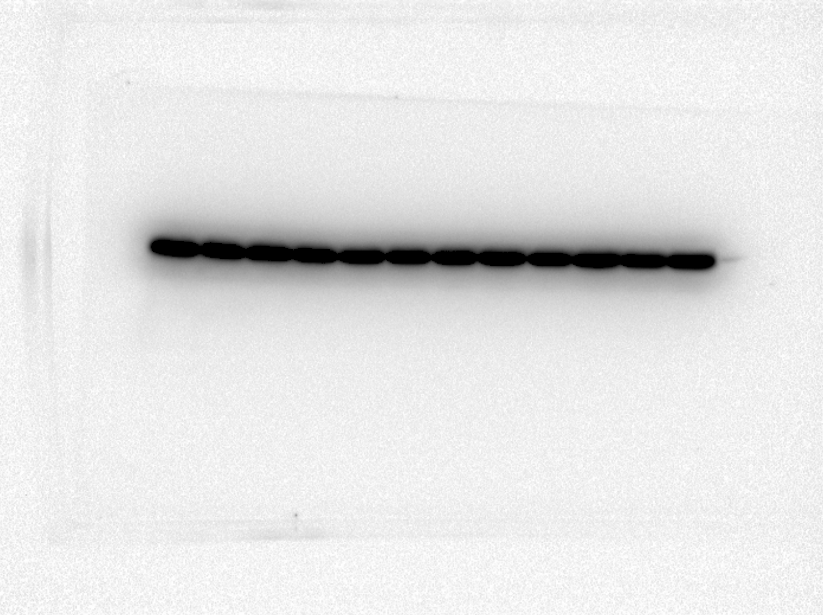


Fig. 5; GAPDH

GAPDH

(37 Kda)

GAPDH

(37 Kda)

GAPDH

(37 Kda)


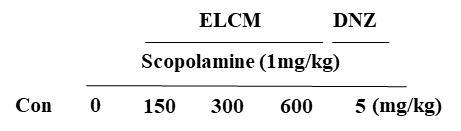

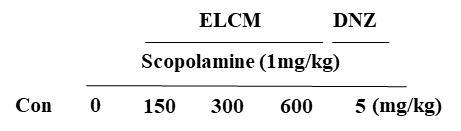

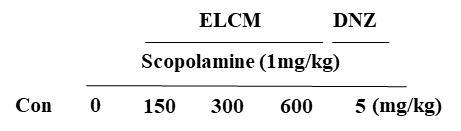

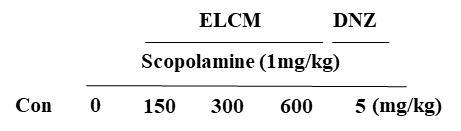

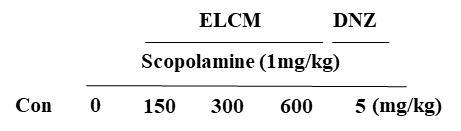

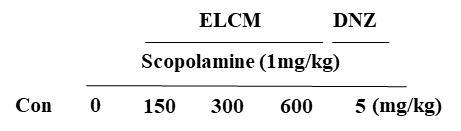


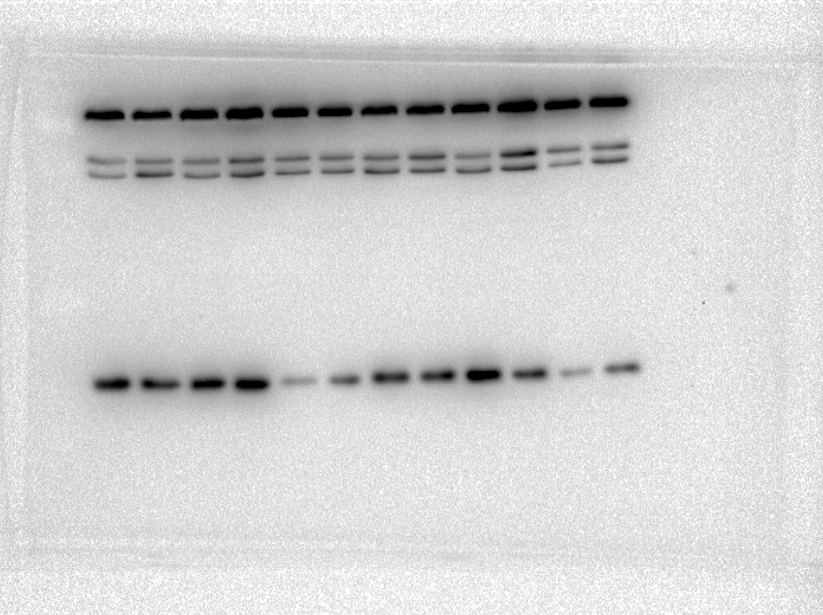

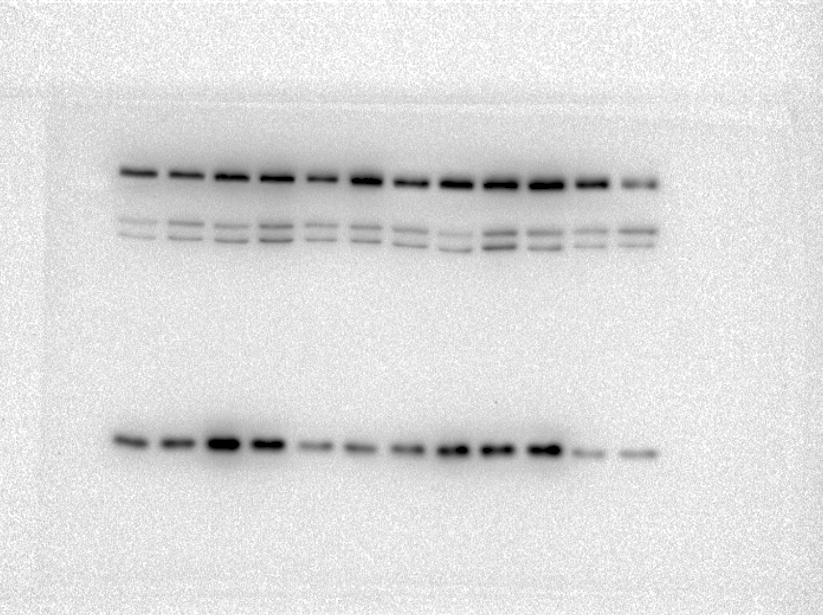

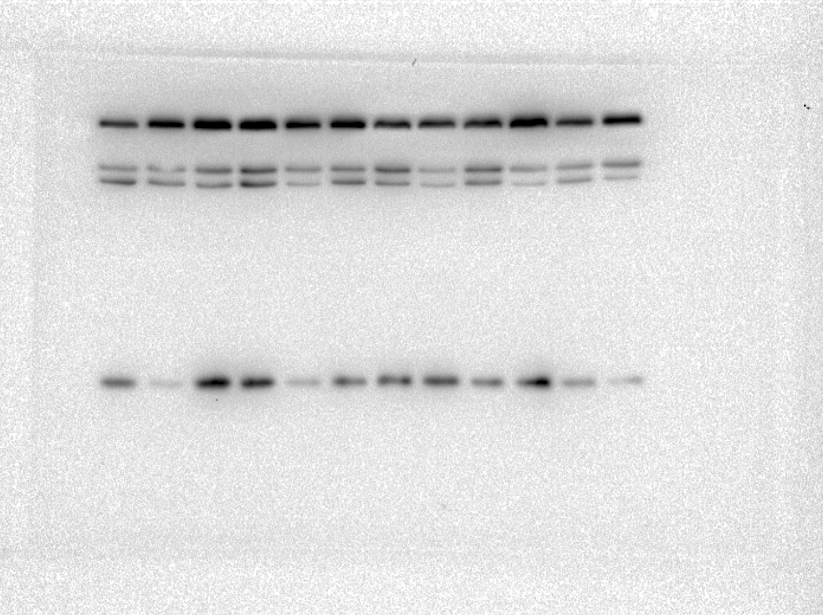


Fig. 6; p-ERK

p-ERK
(44/42 kDa)

p-ERK
(44/42 kDa)

p-ERK
(44/42 kDa)


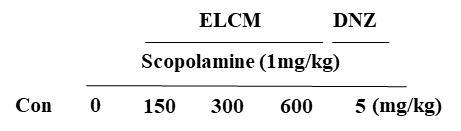

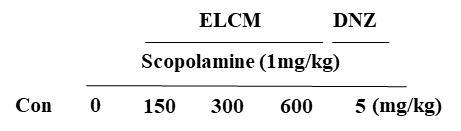

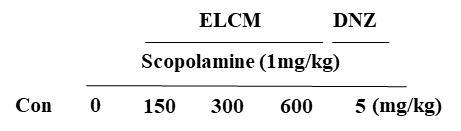

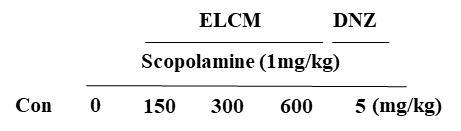

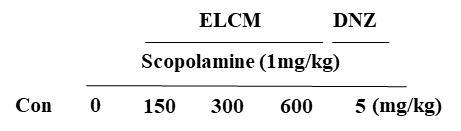

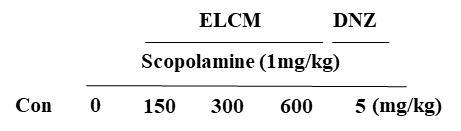


Fig. 6; t-ERK

t-ERK
(43 kDa)

t-ERK
(43 kDa)

t-ERK
(43 kDa)


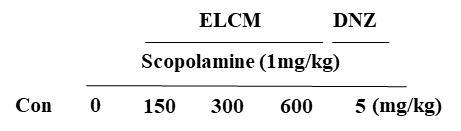

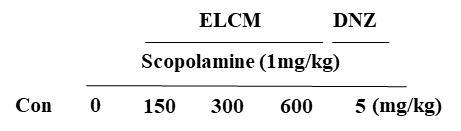

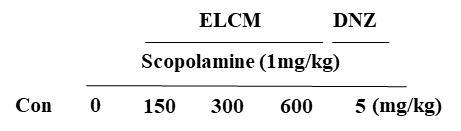

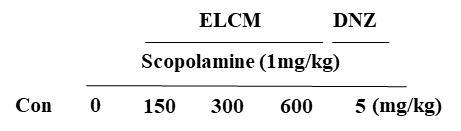

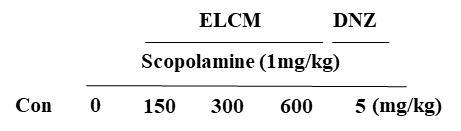

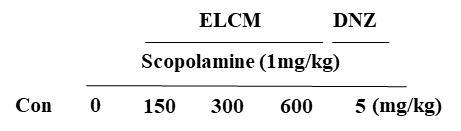


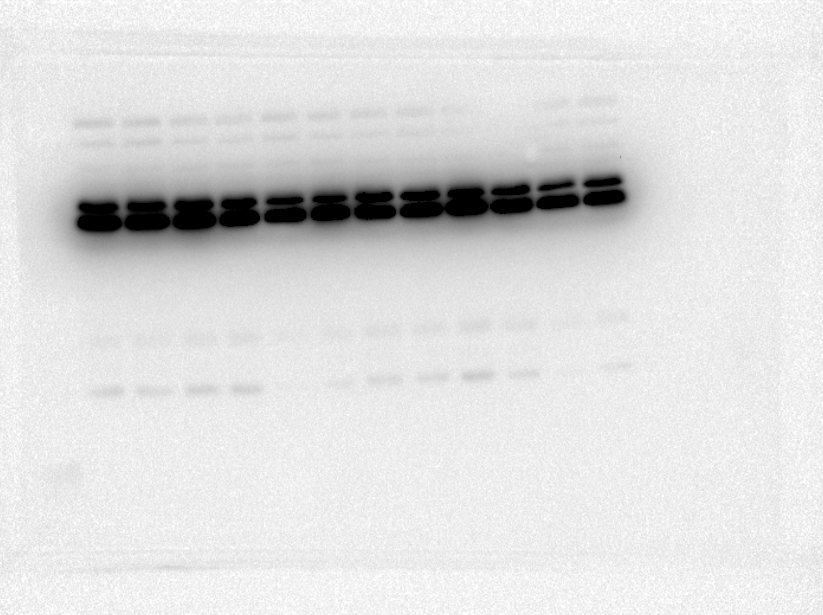

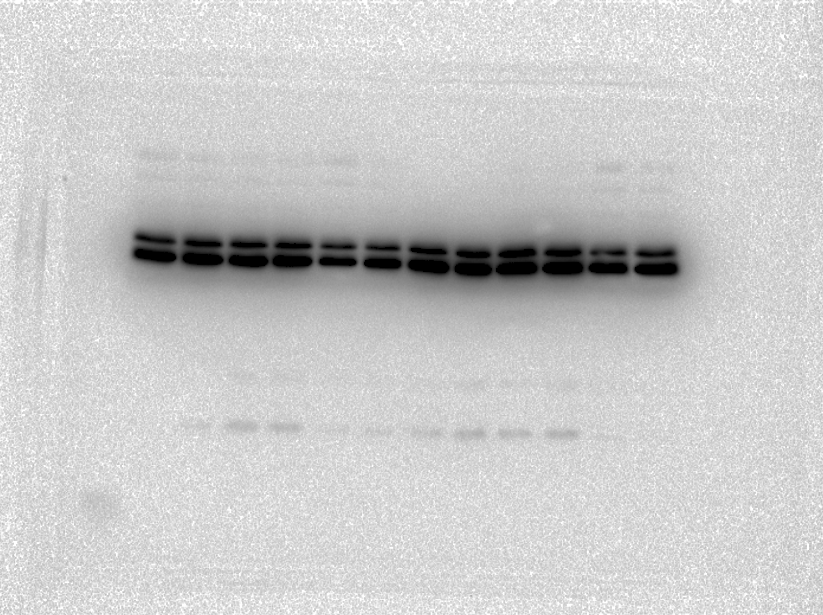

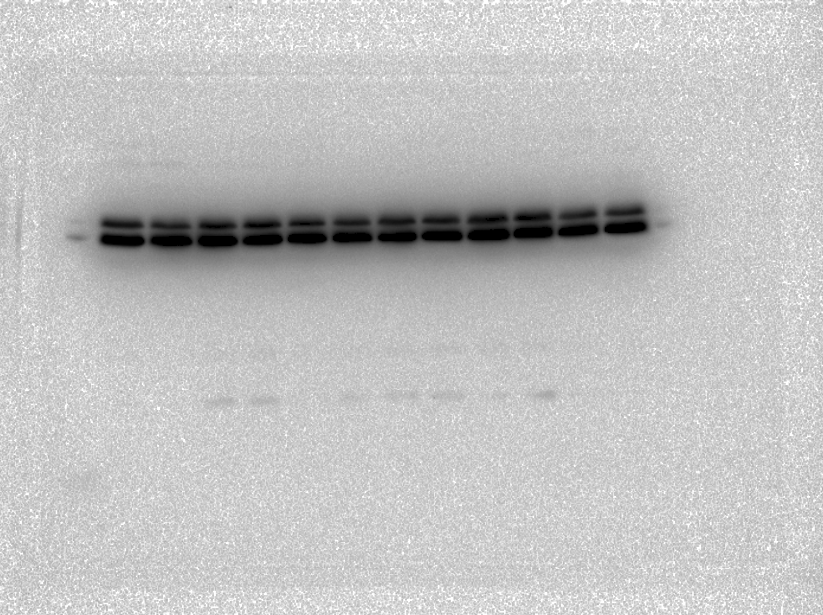


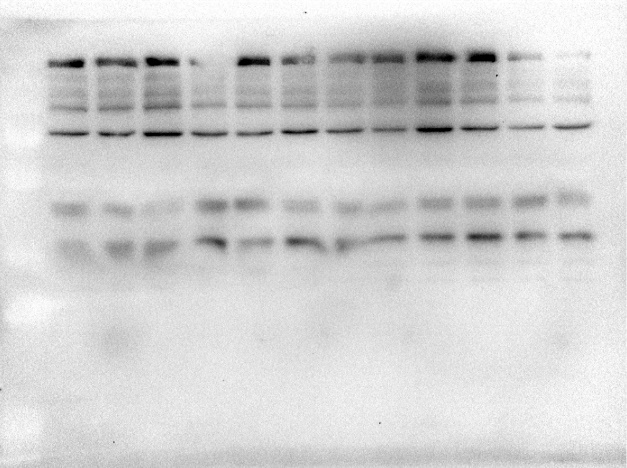

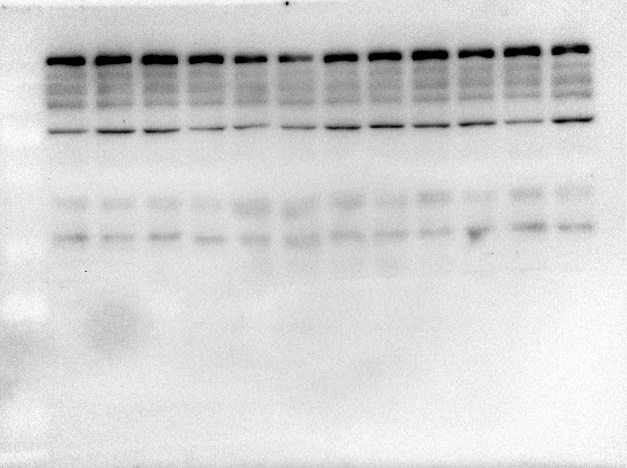

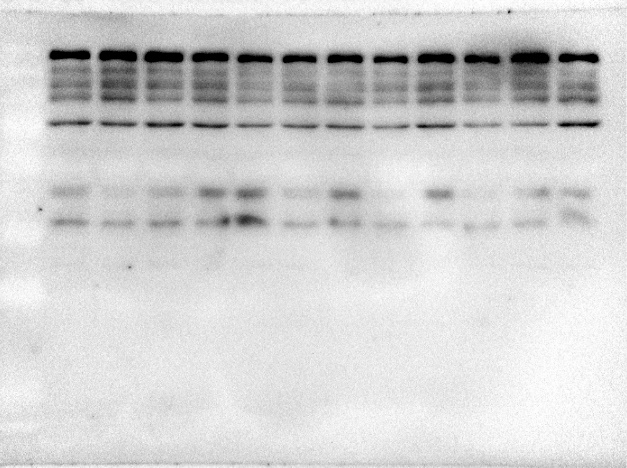


Fig. 6; p-CREB

p-CREB
(45 kDa)

p-CREB
(45 kDa)

p-CREB
(45 kDa)


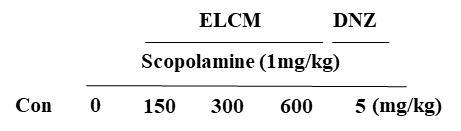

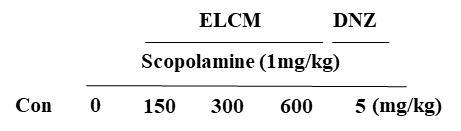

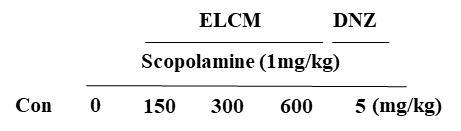

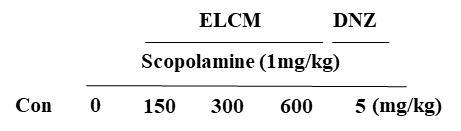

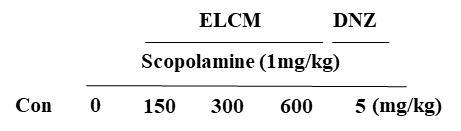

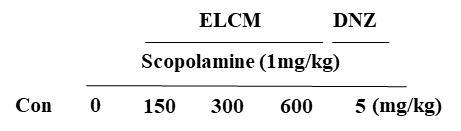


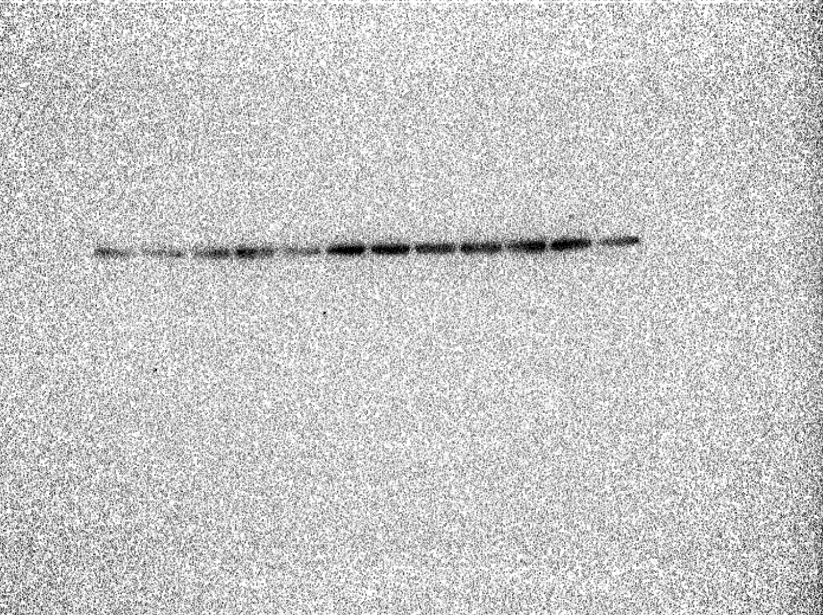

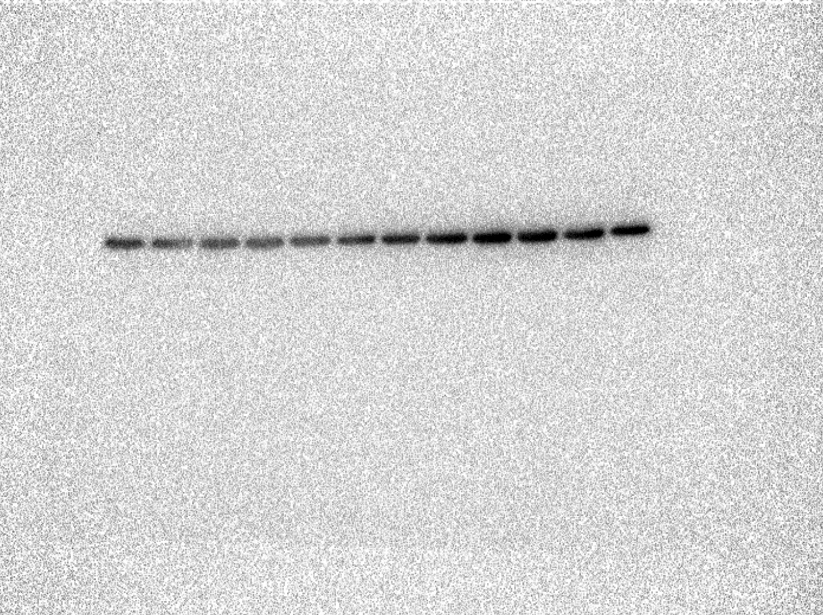

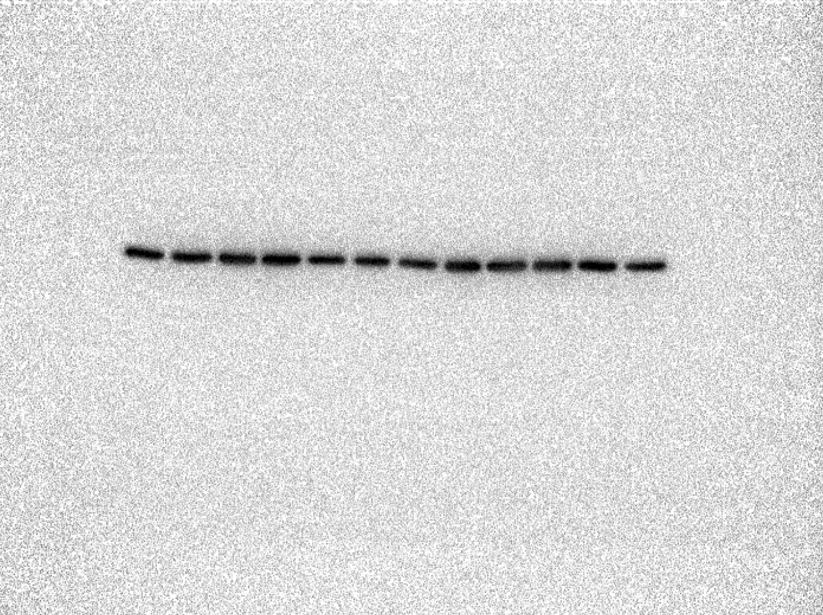


Fig.6; t-CREB

t-CREB
(43 kDa)

t-CREB
(43 kDa)

t-CREB
(43 kDa)


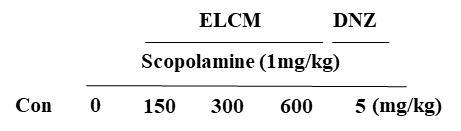

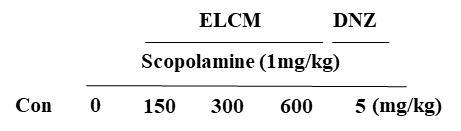

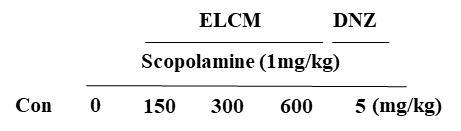

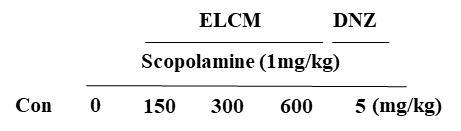

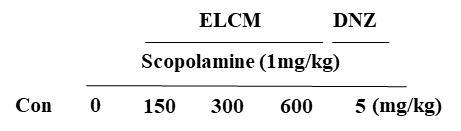

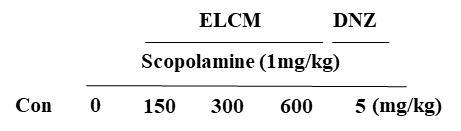


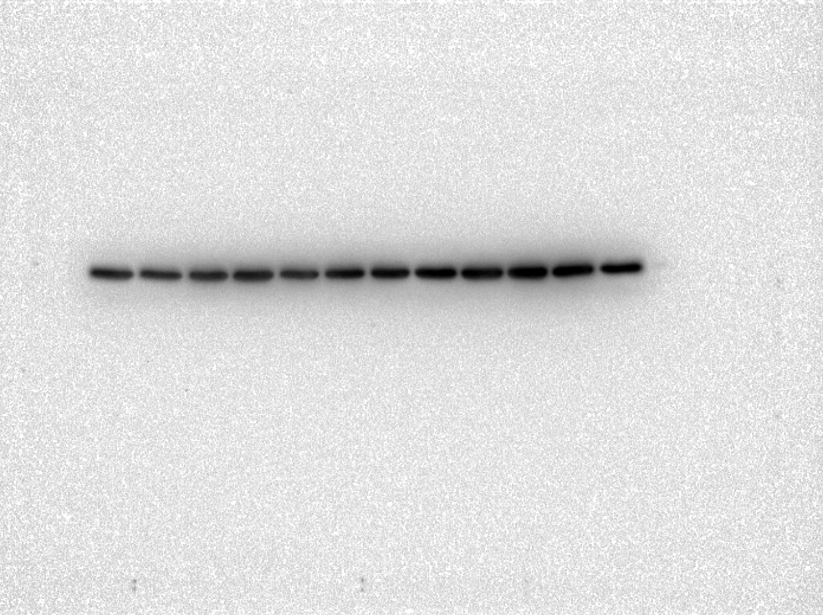


Fig.6; GAPDH

GAPDH
(37 kDa)

GAPDH
(37 kDa)

GAPDH
(37 kDa)
